# Supplementary material for: Multi-landmark alignment of genomic signals reveals conserved expression patterns across transcription start sites
Source: Sci Rep. 2023 Jul 5;13:10835. doi: 10.1038/s41598-023-37140-x (PMC10322939; doi:10.1038/s41598-023-37140-x)
Supplement: Supplementary file 1 — Supplementary Information 1. [file 41598_2023_37140_MOESM1_ESM.pdf]

Multi-landmark alignment in extended genomic-coordinate spaces reveals a conserved pattern of interactions among transcription start sites

Jose M. G. Vilar[1,2,*] *and Leonor Saiz*[3,*]

[1]*Biofisika Intitutua (CSIC, UPV/EHU), University of the Basque Country, P.O. Box 644, 48080 Bilbao, Spain*

[2]*IKERBASQUE, Basque Foundation for Science, 48011 Bilbao, Spain*

[3]*Department of Biomedical Engineering, University of California, 451 East Health Sciences Drive, Davis, CA 95616, USA*

* To whom correspondence should be addressed: j.vilar@ikerbasque.org or lsaiz@ucdavis.edu

SUMMARY: Notebook detailing the code used to compute two-dimensional region averages, as in Supplementary Tables S1 and S2. It downloads the required bigWig signal files from ENCODE and the TSSs from Gencode.

This notebook is provided to ensure reproducibility of the analyses and has not been optimized for general usage. It was executed with python 3.8.5 from the conda 4.9.2 (https://anaconda.org/) distribution with the additional packages pybigwig 0.3.17 and gtfparse 1.2.1.

The main function is get_region_averages(AC_experiment, AC_plus_strand, AC_minus_strand) where the parameters are as follows:

- AC_experiment: ENCODE accession number of the experiment
- AC_plus_strand: ENCODE accession number of BigWig file for the plus strand
- AC_minus_strand: ENCODE accession number of BigWig file for the minus strand

It returns a Pandas dataframe with the data for the five regions, which is also saved as an Excel file named from the concatenation of accesion codes. The notebook computes explcitly the first set of data of Tables S1 and S2.

In [1]:

```
%matplotlib inline
import pandas as pd
import numpy as np
import numpy.ma as ma
import urllib, os
import requests, json
import pickle
import pyBigWig
from gtfparse import read_gtf
```

In [2]:

```
if not os.path.isfile('./GRCh37_p13_v19_TSS.pkl'):
    ofile = "./gencode.v19.annotation.gtf.gz"
    urld = "ftp://ftp.ebi.ac.uk/pub/databases/gencode/Gencode_human/release_19/gencode.v19.annotation.gtf.gz"
    if not os.path.exists(ofile):
        urllib.request.urlretrieve(urld, ofile)

    df = read_gtf("./gencode.v19.annotation.gtf.gz")
    df.gene_id = df.gene_id.str.split(".", expand=True).loc[:, 0]
    df_genes = df[df["feature"] == "gene"]
    df_transcripts = df[(df["feature"] == "transcript")]

    dfg = df_transcripts.groupby("gene_id")
    infodf = pd.DataFrame(
        [
            [
                i.gene_id,
                i.seqname[:],
                i.strand,
                i.start,
                i.end,
                np.sort((dfg.get_group(i.gene_id)["start"]).unique())
                if i.strand == '+' else np.sort(
                    (dfg.get_group(i.gene_id)["end"]).unique())[::-1],
                i.gene_type
            ] for _, i in df_genes.iterrows()
        ][:],
        columns=("gene_id", "seqname", "strand", "start", "end", "tss",
                 "transcript_type"))
    infodf.to_pickle("./GRCh37_p13_v19_TSS.pkl")
```

In [3]:

```
infodf=pd.read_pickle("./GRCh37_p13_v19_TSS.pkl")

protein_coding_genes=infodf[infodf.transcript_type == 'protein_coding'].gene_id
print(len(protein_coding_genes))

def getinfobyid(name):
    try:
        return list(infodf[infodf.gene_id==name].iloc[0,:-1])
```

```
    except:
        print(name, end=' ')
        return [name,"","",0,0,[]]
```

20345

In [4]:
```
def get_aligned_interval_and_distances(info, bwhp, bwhm, tssx=list(range(0, 1)), ltss=1):
    dis1 = []
    dis2 = []
    val = []
    for ii in tssx:
        for name, contig, strand, start, end, n_tss1 in info:
            if len(n_tss1) >= 1 + ii + ltss:
                di = 1 if strand == "+" else -1
                bwh = bwhp if strand == "+" else bwhm
                off2 = (n_tss1[1 + ii] - n_tss1[0 + ii]
                        ) * di if len(n_tss1) >= 2 + ii else np.nan
                off1 = (n_tss1[-1 + ii] - n_tss1[0 + ii]
                        ) * di if ii > 0 else np.nan
                try:
                    valt = (bwh.values(
                        contig, n_tss1[0 + ii] - 2000,
                        n_tss1[0 + ii] + 2000 + 1)[::di])[:]
                    valt = np.nan_to_num(valt)
                    val.append(valt)
                    dis1.append(off1)
                    dis2.append(off2)
                except:
                    print(name, end=' ')
    dis1 = np.array(dis1)
    dis2 = np.array(dis2)
    val = ma.masked_invalid(np.array(val))
    #print ""
    return dis1, dis2, val
```

In [5]:
```
def get_region_averages(a_exp, a_plus, a_minus):

    todfl=[]

    def mxmn(x):
        return min(max(x, 0), 4001)


    headers = {'accept': 'application/json'}
    url = 'https://www.encodeproject.org/experiment/'+a_exp+'/?frame=embedded'
    response = requests.get(url, headers=headers)
    experiment = response.json()

    fir=[fi for fi in experiment['files'] if fi['accession']==a_plus][0]
    fbr,ftr=fir['biological_replicates'],fir['technical_replicates']
    a_gen=[fi['accession'] for fi in experiment['files'] if fi['output_type'] == "gene quantifications" and fi['file_type'] == 'ts

    u_plus="https://www.encodeproject.org/files/" + a_plus + "/@@download/" + a_plus + ".bigWig"
    u_minus="https://www.encodeproject.org/files/" + a_minus + "/@@download/" + a_minus + ".bigWig"
    f_plus="./"+ a_plus + ".bigWig"
    f_minus="./"+ a_minus + ".bigWig"
    urllib.request.urlretrieve(u_plus, f_plus)
    urllib.request.urlretrieve(u_minus, f_minus)
    bwop = pyBigWig.open(f_plus)
    bwom = pyBigWig.open(f_minus)

    u_gen="https://www.encodeproject.org/files/" + a_gen + "/@@download/" + a_gen + ".tsv"
    if experiment['assay_term_name'] == 'RNA-seq':
        dfG = pd.read_table(u_gen, usecols=(0, 6))
    if experiment['assay_term_name'] == 'RAMPAGE':
        dfG = pd.read_table(u_gen, usecols=(6, 7),header=None,names=('FPKM','gene_id'))
        dfG=dfG.groupby("gene_id").sum()
        dfG.reset_index(level=0, inplace=True)

    dfG['gene_id'] = [
        i.split('.')[0] if i.split('.')[:-1] != [] else i
        for i in dfG['gene_id']
    ]

    geneset_temp = dfG.sort_values('FPKM', ascending=False)[:].reset_index(drop=True)
    geneset = geneset_temp[geneset_temp['gene_id'].isin(protein_coding_genes)].reset_index(drop=True)  #is protein

    ind = [
        geneset[geneset['FPKM'] / geneset['FPKM'].mean() < ttii].index[0]
        for ttii in (1e10, 3, 1, 1.0 / 3, 1.0 / 9, 1e-20)
    ][:]

    geneset_temp = dfG.sort_values('FPKM', ascending=False)[:].reset_index(drop=True)
    geneset = geneset_temp[geneset_temp['gene_id'].isin(protein_coding_genes)].reset_index(drop=True)  #is protein

    ind = [
```

```python
        geneset[geneset['FPKM'] / geneset['FPKM'].mean() < ttii].index[0]
        for ttii in (1e10, 3, 1, 1.0 / 3, 1.0 / 9, 1e-20)
    ][:]
    indd = list(zip(ind[:-1], ind[1:]))
    print(indd)
    for AA, i in zip([geneset[i0:i1] for i0, i1 in indd], list(range(5))):
        explev = i
        info = [getinfobyid(i) for i in list(AA['gene_id'])]

        print("\n", AA['FPKM'].mean(axis=0))

        scl = (bwop.header()['sumData']+bwom.header()['sumData'])*1.0/bwop.header()['nBasesCovered']

        for ktss in range(0, 10):

            ticsi = []
            plotsi = []

            _, dis, var = get_aligned_interval_and_distances(info, bwop, bwom,
                                                              np.arange(0, 1 + 0) + ktss, 1)
            dism, _, val = get_aligned_interval_and_distances(info, bwop, bwom,
                                                               np.arange(1, 2 + 0) + ktss, 0)

            disd = np.ma.concatenate([-dism, -dis])
            vald = np.ma.concatenate([val / scl, var / scl])

            ira = np.arange(-400, 1000)
            discc = -disd
            for cc1, cc2 in [(-10000, -1e10), (-200, -900), (200, 0),
                             (1000, 300), (1e10, 10000)]:
                valcc = 1.0 * vald
                for i, d in enumerate(discc):
                    valcc[i, mxmn(2001 + d + cc1 + 1):] = np.nan
                    valcc[i, :mxmn(2000 + d + cc2)] = np.nan
                valcca = np.nanmean(valcc[:, 2000 + ira], axis=0)
                plotsi.append(valcca)
                ticsi.append(
                    np.ma.masked_invalid(
                        np.nanmean(
                            valcc[:, 2000 + 0:2001 + 200],
                            axis=0)).mean())

                if (cc1, cc2)==(-200, -900) or (cc1, cc2)==(-10000, -1e10):
                    ticsi.append(
                        np.ma.masked_invalid(
                            np.nanmean(
                                valcc[:, 2000 + 300:2001 + 1000],
                                axis=0)).mean())

            todfl.append([explev, ktss,] + ticsi)

    aot=pd.DataFrame(todfl, columns=["Transcriptional activity","TSS pair", "T_A","T_Af","T_B","T_Bf","T_C","T_D","T_E"])
    aot["TSS pair"]+=1
    aot["Transcriptional activity"]=aot["Transcriptional activity"].replace({0:"High",1:"Medium-high",2:"Medium",3:"Medium-low",4:
    aot.to_excel("./"+a_exp+a_plus+a_minus+".xlsx",index=False)
    return aot
```

In [6]:

```python
RNAseq001=get_region_averages('ENCSR580GSX','ENCFF233KAB', 'ENCFF028JMF')
RNAseq001
```

```
[(0, 1184), (1184, 3700), (3700, 7355), (7355, 10037), (10037, 14342)]

 323.5647972972975

 49.16017090620047

 17.682429548563615

 6.195906040268462

 0.9030150987224281
```

Out[6]:

	Transcriptional activity	TSS pair	T_A	T_Af	T_B	T_Bf	T_C	T_D	T_E
**0**	High	1	2.305286	1.093601	1.581641	0.434746	10.569687	6.062480	3.745267
**1**	High	2	3.266894	0.516792	4.026818	2.018529	9.714518	6.179036	2.668095
**2**	High	3	5.178540	0.461788	3.560706	2.080112	9.696216	8.089358	4.606993
**3**	High	4	3.595788	0.361176	7.257483	4.175323	9.703165	4.704203	3.627451
**4**	High	5	2.896810	0.281118	6.192476	4.078599	9.384992	6.915636	8.514419
**5**	High	6	3.719979	0.208387	8.563917	2.141968	9.607810	11.272855	4.218151
**6**	High	7	3.942018	0.234043	7.264221	3.312039	10.422291	7.488682	4.905102
**7**	High	8	2.896100	0.861672	8.936675	5.024361	9.104050	6.010517	3.917650
**8**	High	9	4.296502	0.271161	7.048873	1.456201	8.590942	4.522730	4.006766
**9**	High	10	2.574902	0.068225	4.078762	2.349050	8.816354	7.863127	4.063330
**10**	Medium-high	1	0.806037	0.453756	0.350278	0.441464	2.469498	1.991533	1.298163
**11**	Medium-high	2	1.542203	0.324948	1.186758	0.586214	2.510390	1.711857	1.419005
**12**	Medium-high	3	1.850846	0.188490	1.423757	0.549436	2.474080	1.485762	1.455643
**13**	Medium-high	4	1.533765	0.183380	1.278408	0.441868	2.406169	1.330757	1.357586
**14**	Medium-high	5	1.452888	0.135428	1.538445	0.575818	2.276907	1.195491	1.311177
**15**	Medium-high	6	1.268459	0.210244	1.470454	0.490090	2.185313	1.269197	1.413602
**16**	Medium-high	7	1.511813	0.184042	1.259349	0.518424	2.094560	1.229959	1.193612
**17**	Medium-high	8	1.316748	0.202779	1.314685	0.586986	1.837246	1.268565	1.109460
**18**	Medium-high	9	1.101025	0.145551	1.135022	0.618729	1.848875	1.293688	1.148809
**19**	Medium-high	10	1.237007	0.213245	1.086449	0.630723	1.633153	1.398593	1.079080
**20**	Medium	1	0.589067	0.162749	0.225718	0.321566	0.934962	0.778292	0.535979
**21**	Medium	2	0.723380	0.140922	0.495265	0.253204	0.942224	0.636521	0.588878
**22**	Medium	3	0.713145	0.073489	0.534379	0.245041	0.909920	0.493049	0.566291
**23**	Medium	4	0.637892	0.101930	0.561522	0.269126	0.851487	0.555723	0.502380
**24**	Medium	5	0.558075	0.097355	0.568633	0.192271	0.800569	0.569928	0.542117
**25**	Medium	6	0.570551	0.081100	0.559632	0.234692	0.737675	0.489110	0.540728
**26**	Medium	7	0.505146	0.082421	0.439960	0.254996	0.706714	0.541398	0.454595
**27**	Medium	8	0.495712	0.101197	0.467203	0.292915	0.668136	0.527828	0.407871
**28**	Medium	9	0.492673	0.091624	0.476997	0.258547	0.594542	0.518307	0.424382
**29**	Medium	10	0.361411	0.114622	0.463223	0.279099	0.597600	0.466674	0.409442
**30**	Medium-low	1	0.328610	0.105231	0.084858	0.101722	0.361433	0.260717	0.238371
**31**	Medium-low	2	0.299498	0.062385	0.208157	0.132874	0.336139	0.238788	0.206795
**32**	Medium-low	3	0.253057	0.051720	0.226857	0.098504	0.325023	0.216718	0.205218
**33**	Medium-low	4	0.233476	0.043014	0.218028	0.107513	0.294570	0.218914	0.197534
**34**	Medium-low	5	0.190474	0.039134	0.184527	0.083989	0.283768	0.184312	0.182501
**35**	Medium-low	6	0.203280	0.036472	0.174567	0.087740	0.270258	0.173733	0.163465
**36**	Medium-low	7	0.168411	0.034409	0.164969	0.108408	0.247469	0.190284	0.166583
**37**	Medium-low	8	0.216826	0.046057	0.180304	0.089244	0.224675	0.190501	0.161036
**38**	Medium-low	9	0.150078	0.042699	0.139516	0.099889	0.190915	0.174049	0.127962
**39**	Medium-low	10	0.137592	0.030113	0.148880	0.062913	0.181337	0.131238	0.141731
**40**	Low	1	0.121916	0.024900	0.044002	0.031313	0.099842	0.060071	0.043094
**41**	Low	2	0.058189	0.010280	0.040080	0.020948	0.078519	0.050488	0.038025
**42**	Low	3	0.050445	0.011768	0.045892	0.021833	0.061414	0.038378	0.032340
**43**	Low	4	0.030649	0.006713	0.033859	0.017375	0.052065	0.029856	0.029897
**44**	Low	5	0.040013	0.009826	0.028282	0.020350	0.044987	0.024678	0.027565
**45**	Low	6	0.030994	0.008001	0.034763	0.012394	0.038764	0.026502	0.030059
**46**	Low	7	0.029146	0.006358	0.020242	0.018502	0.035851	0.031265	0.024766
**47**	Low	8	0.029558	0.005137	0.025509	0.015576	0.035267	0.030055	0.024380

	Transcriptional activity	TSS pair	T_A	T_Af	T_B	T_Bf	T_C	T_D	T_E
**48**	Low	9	0.025596	0.007659	0.025434	0.015669	0.027146	0.027324	0.023561
**49**	Low	10	0.021627	0.004330	0.023702	0.016582	0.026994	0.024023	0.030391

In [7]:
```
RAMPAGE001=get_region_averages('ENCSR208AWA','ENCFF733LTT', 'ENCFF699MEA')
RAMPAGE001
```

```
[(0, 1076), (1076, 2313), (2313, 4032), (4032, 5665), (5665, 10573)]

 7246.758364312268

 827.3961196443007

 279.40314136125653

 94.03368034292713

 13.292583537082315
```

Out[7]:

	Transcriptional activity	TSS pair	T_A	T_Af	T_B	T_Bf	T_C	T_D	T_E
**0**	High	1	0.031442	0.000145	0.041797	0.000186	0.630718	0.473270	0.455242
**1**	High	2	0.049486	0.000114	0.089303	0.000126	0.454052	0.659307	0.123931
**2**	High	3	0.031209	0.000051	0.065208	0.000372	0.386061	0.656028	0.910785
**3**	High	4	0.031030	0.000028	0.142587	0.001844	0.351095	0.109593	0.034382
**4**	High	5	0.035646	0.000076	0.129957	0.000987	0.311410	0.146684	0.000908
**5**	High	6	0.017390	0.000080	0.025068	0.000294	0.259545	0.023072	0.012890
**6**	High	7	0.008643	0.000030	0.037012	0.001095	0.158770	0.009715	0.016575
**7**	High	8	0.003694	0.000122	0.023971	0.001472	0.120758	0.001829	0.000921
**8**	High	9	0.003326	0.000027	0.010002	0.000293	0.114353	0.009249	0.000612
**9**	High	10	0.002481	0.000032	0.009975	0.000461	0.084308	0.004250	0.007907
**10**	Medium-high	1	0.075341	0.000076	0.010472	0.000612	0.088481	0.091253	0.070342
**11**	Medium-high	2	0.045938	0.000763	0.031959	0.000155	0.065261	0.061192	0.014630
**12**	Medium-high	3	0.027709	0.000066	0.013852	0.000654	0.047189	0.034680	0.007905
**13**	Medium-high	4	0.012034	0.000056	0.021527	0.000081	0.034405	0.019740	0.004404
**14**	Medium-high	5	0.005424	0.000061	0.009160	0.002293	0.028563	0.015955	0.002978
**15**	Medium-high	6	0.005453	0.000029	0.006646	0.000112	0.023848	0.006943	0.002591
**16**	Medium-high	7	0.002989	0.000035	0.005909	0.000135	0.018840	0.011409	0.001297
**17**	Medium-high	8	0.002748	0.000042	0.004611	0.000093	0.016001	0.001325	0.000184
**18**	Medium-high	9	0.003344	0.000035	0.001833	0.000168	0.012105	0.000674	0.000528
**19**	Medium-high	10	0.000728	0.000035	0.000944	0.000186	0.010533	0.002564	0.000230
**20**	Medium	1	0.017652	0.000083	0.007928	0.000062	0.033081	0.035963	0.024922
**21**	Medium	2	0.018513	0.000037	0.011029	0.000248	0.024394	0.026609	0.014079
**22**	Medium	3	0.007893	0.000032	0.013116	0.000725	0.016544	0.013504	0.006202
**23**	Medium	4	0.004384	0.000048	0.005853	0.000904	0.012423	0.008236	0.002043
**24**	Medium	5	0.001963	0.000030	0.004233	0.000143	0.009308	0.003409	0.001761
**25**	Medium	6	0.001932	0.000040	0.004959	0.000292	0.007421	0.002695	0.002187
**26**	Medium	7	0.002007	0.000037	0.004214	0.000121	0.006048	0.001586	0.000548
**27**	Medium	8	0.000893	0.000060	0.001861	0.000097	0.004648	0.001232	0.000215
**28**	Medium	9	0.000589	0.000015	0.000997	0.000102	0.003816	0.001252	0.000965
**29**	Medium	10	0.000574	0.000025	0.000770	0.000323	0.003437	0.000453	0.001755
**30**	Medium-low	1	0.015786	0.000068	0.019166	0.000177	0.012671	0.016888	0.005162
**31**	Medium-low	2	0.006446	0.000047	0.004704	0.000408	0.012109	0.006498	0.003102
**32**	Medium-low	3	0.004579	0.000026	0.007383	0.000088	0.008964	0.005523	0.001263
**33**	Medium-low	4	0.002133	0.000023	0.002934	0.000085	0.005088	0.003368	0.000874
**34**	Medium-low	5	0.001943	0.000020	0.002372	0.000102	0.003910	0.002020	0.000356
**35**	Medium-low	6	0.001600	0.000043	0.001374	0.000091	0.002892	0.001198	0.000545
**36**	Medium-low	7	0.000452	0.000014	0.000685	0.000059	0.002221	0.000522	0.000600
**37**	Medium-low	8	0.000399	0.000078	0.000386	0.000044	0.002004	0.000674	0.000481
**38**	Medium-low	9	0.000357	0.000025	0.000736	0.000057	0.001779	0.000350	0.000325
**39**	Medium-low	10	0.000553	0.000021	0.000210	0.000033	0.001580	0.001096	0.000284
**40**	Low	1	0.006883	0.000288	0.003631	0.000847	0.002675	0.002826	0.001743
**41**	Low	2	0.002019	0.000165	0.002675	0.000482	0.002084	0.001453	0.001994
**42**	Low	3	0.000703	0.000020	0.001431	0.000149	0.002112	0.001307	0.000235
**43**	Low	4	0.000371	0.000020	0.001411	0.000066	0.001974	0.001290	0.000251
**44**	Low	5	0.000455	0.000020	0.001293	0.000124	0.000985	0.000694	0.000233
**45**	Low	6	0.000197	0.000017	0.000502	0.000042	0.000911	0.000462	0.000191
**46**	Low	7	0.000261	0.000020	0.000292	0.000050	0.000710	0.000334	0.000504
**47**	Low	8	0.000303	0.000017	0.000808	0.000042	0.000613	0.000210	0.000156

	Transcriptional activity	TSS pair	T_A	T_Af	T_B	T_Bf	T_C	T_D	T_E
**48**	Low	9	0.000155	0.000016	0.000135	0.000057	0.000547	0.000227	0.000173
**49**	Low	10	0.000109	0.000013	0.000464	0.000049	0.000394	0.000213	0.000218

In [ ]:
