# Supplementary material for: Multi-landmark alignment of genomic signals reveals conserved expression patterns across transcription start sites
Source: Sci Rep. 2023 Jul 5;13:10835. doi: 10.1038/s41598-023-37140-x (PMC10322939; doi:10.1038/s41598-023-37140-x)
Supplement: Supplementary file 2 — Supplementary Information 2. [file 41598_2023_37140_MOESM2_ESM.pdf]

Multi-landmark alignment in extended genomic-coordinate spaces reveals a conserved pattern of interactions among transcription start sites

Jose M. G. Vilar[1,2,*] *and Leonor Saiz*[3,*]

[1]*Biofisika Intitutua (CSIC, UPV/EHU), University of the Basque Country, P.O. Box 644, 48080 Bilbao, Spain*

[2]*IKERBASQUE, Basque Foundation for Science, 48011 Bilbao, Spain*

[3]*Department of Biomedical Engineering, University of California, 451 East Health Sciences Drive, Davis, CA 95616, USA*

* To whom correspondence should be addressed: j.vilar@ikerbasque.org or lsaiz@ucdavis.edu

SUMMARY: Notebook to compute two-dimensional signal densities, as in Figures 2, 3, and 4. It downloads the required bigWig signal files from ENCODE and the TSSs from Gencode.

This notebook is provided to ensure reproducibility of the analyses and has not been optimized for general usage. It was executed with python 3.8.5 from the conda 4.9.2 (https://anaconda.org/) distribution with the additional packages pybigwig 0.3.17 and gtfparse 1.2.1.

In [1]:
```
%matplotlib inline
import pandas as pd
import numpy as np
import numpy.ma as ma
import matplotlib.pyplot as plt
import matplotlib.patches as patches
from scipy.interpolate import griddata
import pyBigWig
from gtfparse import read_gtf
import urllib,os
```

In [2]:
```
plt.style.use('classic')
```

In [3]:
```
plt.rcParams['ps.fonttype'] = 3
plt.rcParams['pdf.fonttype'] = 42
plt.rcParams['lines.linewidth'] = 2
plt.rcParams['axes.linewidth'] = 1.0
plt.rcParams['font.size'] = 16
plt.rcParams['font.weight'] = 'light'
plt.rcParams['font.style'] = 'normal'
plt.rcParams['mathtext.fontset'] = 'stix'
```

In [4]:
```
if not os.path.isfile('./GRCh37_p13_v19_TSS.pkl'):
    ofile = "./gencode.v19.annotation.gtf.gz"
    urld = "ftp://ftp.ebi.ac.uk/pub/databases/gencode/Gencode_human/release_19/gencode.v19.annotation.gtf.gz"
    if not os.path.exists(ofile):
        urllib.request.urlretrieve(urld, ofile)

    df = read_gtf("./gencode.v19.annotation.gtf.gz")
    df.gene_id = df.gene_id.str.split(".", expand=True).loc[:, 0]
    df_genes = df[df["feature"] == "gene"]
    df_transcripts = df[(df["feature"] == "transcript")]

    dfg = df_transcripts.groupby("gene_id")
    infodf = pd.DataFrame(
        [
            [
                i.gene_id,
                i.seqname[:],
                i.strand,
                i.start,
                i.end,
                np.sort((dfg.get_group(i.gene_id)["start"]).unique())
                if i.strand == '+' else np.sort(
                    (dfg.get_group(i.gene_id)["end"]).unique())[::-1],
                i.gene_type
            ] for _, i in df_genes.iterrows()
        ][:],
        columns=("gene_id", "seqname", "strand", "start", "end", "tss",
                 "transcript_type"))
    infodf.to_pickle("./GRCh37_p13_v19_TSS.pkl")
```

In [5]:
```
infodf = pd.read_pickle("./GRCh37_p13_v19_TSS.pkl")

protein_coding_genes = infodf[infodf.transcript_type == 'protein_coding'].gene_id
print(len(protein_coding_genes))
```

```python
def getinfobyid(name):
    try:
        return list(infodf[infodf.gene_id == name].iloc[0, :-1])
    except:
        print(name, end=' ')
        return [name, "", "", 0, 0, []]
```

20345

In [6]:

```python
def get_aligned_interval_and_distances(info, bwhp, bwhm, tssx=list(range(0, 1)), ltss=1):
    dis1 = []
    dis2 = []
    val = []
    for ii in tssx:
        print("****", ii, end=' ')
        for name, contig, strand, start, end, n_tss1 in info:
            if len(n_tss1) >= 1 + ii + ltss:
                di = 1 if strand == "+" else -1
                bwh = bwhp if strand == "+" else bwhm
                off2 = (n_tss1[1 + ii] - n_tss1[0 + ii]
                        ) * di if len(n_tss1) >= 2 + ii else np.nan
                off1 = (n_tss1[-1 + ii] - n_tss1[0 + ii]
                        ) * di if ii > 0 else np.nan
                try:
                    valt = (bwh.values(
                        contig, n_tss1[0 + ii] - 2000,
                        n_tss1[0 + ii] + 2000 + 1)[::di])[:]
                    valt = np.nan_to_num(valt)
                    val.append(valt)
                    dis1.append(off1)
                    dis2.append(off2)
                except:
                    print(name, end=' ')
    dis1 = np.array(dis1)
    dis2 = np.array(dis2)
    val = ma.masked_invalid(np.array(val))
    print("")
    return dis1, dis2, val
```

In [7]:

```python
def two_dimensional_average(valp,
                            disp,
                            valm,
                            dism,
                            dd1=90,
                            step1=25,
                            dd2=4000,
                            step2=250):
    xxl, yyl, zzl = [], [], []
    for i in range(-1000 + 1, 1000)[::step1]:
        for j in range(-1000, 1000)[::step1]:
            if j < i:
                idy = (disp <= i - j + 2 * dd1) & (disp >= i - j - 2 * dd1)
                idym = (dism <= i - j + 2 * dd1) & (dism >= i - j - 2 * dd1)
                if False & (len(valp) > 0) & (len(valm) > 0):
                    zzl.append(
                        np.ma.concatenate([
                            valp[idy, 2000 + i - dd1:2000 + i + dd1 + 1],
                            valm[idym, 2000 + j - dd1:2000 + j + dd1 + 1]
                        ]).mean())
                elif (len(valm) > 0) & (j > -i):
                    Idm = dism[idym]
                    nan_valm = 1.0 * valm[idym, 2000 - 2 * dd1 + j:
                                          2000 + 2 * dd1 + j]
                    for iii, iIdm in enumerate(Idm):
                        nan_valm[iii, :2 * dd1 +
                                 max(-dd1 - (iIdm - i + j), -dd1) + 1] = np.nan
                        nan_valm[iii, 2 * dd1 +
                                 min(+dd1 - (iIdm - i + j), +dd1):] = np.nan
                    zzl.append(np.nanmean(nan_valm))
                elif (len(valp) > 0) & (j <= -i):
                    Id = disp[idy]
                    nan_val = 1.0 * valp[idy, 2000 - 2 * dd1 + i:
                                         2000 + 2 * dd1 + i]
                    for iii, iId in enumerate(Id):
                        nan_val[iii, :2 * dd1 + max(-dd1 + (iId - i + j), -dd1) +
                                1] = np.nan
                        nan_val[iii, 2 * dd1 +
                                min(+dd1 + (iId - i + j), +dd1):] = np.nan
                    zzl.append(np.nanmean(nan_val))
                yyl.append(j)
                xxl.append(i)
    dd0 = dd2
    for i in range(-1000, 1000)[::step1]:
        for j in range(-20000, -1000)[::step2]:
            if j < i:
                dd2 = min(-j // 4, dd0)
```

```
                idy = (disp <= i - j + dd2 + dd1) & (disp >= i - j - dd2 - dd1)
                Id = disp[idy]
                nan_val = 1.0 * valp[idy, 2000 - 2 * dd1 + i:2000 + 2 * dd1 + i]
                for iii, iId in enumerate(Id):
                    nan_val[iii, :2 * dd1 + max(-dd2 + (iId - i + j), -dd1) +
                                  1] = np.nan
                    nan_val[iii, 2 * dd1 + min(+dd2 +
                                               (iId - i + j), +dd1):] = np.nan
                zzl.append(np.nanmean(nan_val))
                yyl.append(j)
                xxl.append(i)

    for i in range(1000, 20000 + step2)[::step2]:
        for j in range(-1000, 1000 + step1)[::step1]:
            if j < i:
                dd2 = min(i // 4, dd0)
                idym = (dism <= i - j + dd2 + dd1) & (dism >=
                                                      i - j - dd2 - dd1)
                Idm = dism[idym]
                nan_valm = 1.0 * valm[idym, 2000 - 2 * dd1 + j:
                                      2000 + 2 * dd1 + j]
                for iii, iIdm in enumerate(Idm):
                    nan_valm[iii, :2 * dd1 + max(-dd2 - (iIdm - i + j), -dd1) +
                                   1] = np.nan
                    nan_valm[iii, 2 * dd1 +
                                  min(+dd2 - (iIdm - i + j), +dd1):] = np.nan
                zzl.append(np.nanmean(nan_valm))
                yyl.append(j)
                xxl.append(i)

    xxl = (np.array(xxl)).flatten()
    yyl = (np.array(yyl)).flatten()
    zzl = (np.array(zzl)).flatten()
    return xxl, yyl, zzl
```

In [8]:

```
genesq = './ENCFF782PCD.tsv'
bwhpL = [
    './ENCFF091RAW.bigWig',
    './ENCFF707TAV.bigWig',
    './ENCFF000YWY.bigWig',
    './ENCFF000SVL.bigWig',
    './ENCFF000BYB.bigWig'
]
bwhmL = [
    './ENCFF652ZSN.bigWig',
    './ENCFF198YEH.bigWig',
    './ENCFF000YWY.bigWig',
    './ENCFF000SVL.bigWig',
    './ENCFF000BYB.bigWig'
]
titL = ('RNA', 'RAMPAGE', 'POLR2A', 'DNase', 'H3K4me3')

for ofile in [genesq,]+bwhpL+bwhmL:
    acc=ofile.split(".")[-2].split("/")[-1]
    urld="https://www.encodeproject.org/files/"+acc+"/@@download/"+ofile
    if not os.path.exists(ofile):
        urllib.request.urlretrieve(urld, ofile)



dfG = pd.read_table(
    genesq, usecols=(0, 6))

dfG['gene_id'] = [
    i.split('.')[0] if i.split('.')[:-1] != [] else i for i in dfG['gene_id']
]

geneset = dfG[dfG['gene_id'].isin(protein_coding_genes)].sort_values(
    'FPKM', ascending=False).reset_index(drop=True)  #is protein
```

In [9]:

```
def plot_density(axv, clabel, xxl, yyl, zzl, region=1, textla=""):
    global zim, CSg, CS2g, azim

    x, y, z = xxl, yyl, zzl

    numcols, numrows = 60 * 4, 60 * 4
    LMx = 500
    LMy = 1000
    if region == 1:
        xi = np.linspace(-LMx, 1000, numcols)
        yi = np.linspace(-LMy, 1000, numrows)
    if region == 2:
        xi = np.linspace(-LMx, 1000, numcols)
        yi = np.linspace(-20000, -LMy, numrows)
    if region == 3:
```

```python
        xi = np.linspace(1000, 20000, numcols)
        yi = np.linspace(-LMy, 1000, numrows)
    if region == 4:
        cbar = plt.colorbar(CSg, cax=axv, orientation="horizontal")
        cbar.ax.set_xlabel(clabel)
        cbar.add_lines(CS2g)
        return

    xib = np.linspace(1.5 * xi[0] - 0.5 * xi[1], 1.5 * xi[-1] - 0.5 * xi[-2],
                      numcols + 1)
    yib = np.linspace(1.5 * yi[0] - 0.5 * yi[1], 1.5 * yi[-1] - 0.5 * yi[-2],
                      numrows + 1)

    zi = griddata(
        (x, y), z, (xi[None, :], yi[:, None]), method='linear').astype(float)

    digs = max(0, int(round(1 - np.log10(np.nanmax(zi) / 2.5), 0)))
    vmaxv = round(
        10**-digs / 2.01 + 1.1 * np.nanmax(z[(y - x > -1000) & (x < 900) &
                                              (x > -300) & (y > -800) &
                                              (y < 800)]), digs)

    if region == 1:
        zim = round(10**-digs / 2.01 + np.nanmax(zi), digs)
    zi = zi * (zi < vmaxv) + vmaxv * (zi >= vmaxv)
    zi = zi + 1e-10
    vmaxv, extendv = (vmaxv, 'max') if vmaxv < zim else (zim, 'neither')

    vminv = 0
    extendv = 'max'
    colm = plt.cm.CMRmap_r
    CS = axv.contourf(
        xi,
        yi,
        zi,
        np.arange(vminv, vmaxv + 1e-10, (vmaxv - vminv) / 10),
        cmap=colm,
        extend=extendv,
        zorder=-20)
    CS2 = axv.contour(
        CS, levels=CS.levels[::1], cmap=colm, hold='on', zorder=-15)
    if region == 1:
        CSg, CS2g = CS, CS2
        axv.add_patch(
            patches.Rectangle(
                (-LMx, -LMy),
                LMx + 1000,
                LMy + 1000,
                lw=0,
                color=(0.8, 0.8, 0.85),
                zorder=-25))
        axv.text(-LMx + 70, 700, textla, zorder=5)
    axv.grid(
        b=True, which=['major', 'both'][0], linestyle='-', lw=1, color='white')
    plt.setp(list(axv.spines.values()), zorder=5)
    axv.set_rasterization_zorder(-10)


def composite_plot(tit, xxl, yyl, zzl, xlabel="TSS n", ylabel="TSS n+1", textla=""):
    ox, oy = 0.15, -0.125
    #zim = 10000
    axn = plt.axes([0 + ox, 0.5 + oy, 0.375, 0.5])
    plot_density(axn, tit, xxl, yyl, zzl, 1, textla)
    plt.xticks([-500, 0, 500, 1000])
    axn.xaxis.tick_top()
    axn.set_xticks(axn.get_xticks()[:])
    plt.xticks(rotation=35)
    axn.set_xlabel(xlabel, x=0.65, fontsize=17)
    axn.xaxis.set_label_position('top')
    plt.xlim(-500, 1000)
    plt.ylabel(ylabel, y=0.35, fontsize=17)
    axn2 = plt.axes([0 + ox, 0.3 + oy - 0.005, 0.375, 0.2])
    plot_density(axn2, tit, xxl, yyl, zzl, 2)
    plt.xticks([-500, 0, 500, 1000])
    axn2.set_xticklabels([])
    axn2.set_yticks(axn2.get_yticks()[:-1])
    axn2.set_yticklabels(["−20k", "−15k", "−10k", "−5k"])
    axn3 = plt.axes([0.375 + ox + 0.005, 0.5 + oy, 0.2, 0.5])
    plot_density(axn3, tit, xxl, yyl, zzl, 3)
    axn3.xaxis.tick_top()
    axn3.set_yticklabels([])
    axn3.set_xticks(axn3.get_xticks()[1:])
    axn3.set_xticklabels(["5k", "10k", "15k", "20k"])
    plt.xticks(rotation=35)

    axn4 = plt.axes([0.05 + ox, 0.25 + oy, 0.5, 0.025])
```

```python
    plt.xticks(rotation=35)

    plot_density(axn4, tit, xxl, yyl, zzl, 4)
```

In [10]:
```python
print(geneset['FPKM'].mean(), len(geneset))
ind = [
    geneset[geneset['FPKM'] / geneset['FPKM'].mean() < ttii].index[0]
    for ttii in (1e10, 3, 1, 1.0 / 3, 1.0 / 9, 1e-20)
]
indd = list(zip(ind[:-1], ind[1:]))
print(indd)
```

```
30.518960432539433 20345
[(0, 987), (987, 2955), (2955, 6107), (6107, 8839), (8839, 13641)]
```

In [11]:
```python
from matplotlib.transforms import Bbox


def savefig(figcomp, name):
    for i in ("pdf", ):
        figcomp.savefig(
            "./" + name + "." + i,
            transparent=True,
            dpi=75,
            bbox_inches=Bbox([[-0.25, -0.25], [7.5,
                                               8]]))  #,bbox_inches='tight')
```

In [12]:
```python
textlatl = [
    "High\ntranscription", "Medium-high\ntranscription",
    "Medium\ntranscription", "Medium-low\ntranscription", "Low\ntranscription"
]

for kk in (0, 1):
    for AA, ix, textlat in list(zip([geneset[i0:i1] for i0, i1 in indd],
                               [i1 for i0, i1 in indd], textlatl))[::2]:
        info = [getinfobyid(i) for i in list(AA['gene_id'])]

        for bwhpn, bwhmn, tit in list(zip(bwhpL, bwhmL, titL))[:]:
            bwop = pyBigWig.open(bwhpn)  ###
            bwom = pyBigWig.open(bwhmn)  ###
            scl = (
                bwop.header()['sumData'] * 1.0 / bwop.header()['nBasesCovered']
                if tit != "RNA" and tit != "RAMPAGE" else
                (bwop.header()['sumData'] + bwom.header()['sumData']) * 1.0 / bwop.header()['nBasesCovered']
            )
            print(scl)

            print("{}_{}_{}_{}".format("K562", kk, ix, tit))
            _, dis, var = get_aligned_interval_and_distances(info, bwop, bwom, np.arange(0, 1) + kk, 1)
            dism, _, val = get_aligned_interval_and_distances(info, bwop, bwom, np.arange(1, 2) + kk, 0)
            xxl, yyl, zzl = two_dimensional_average(var / scl, dis, val / scl, -dism, 99, 25)
            figcomp = plt.figure(figsize=(7 + 2 - 1.5, 5.5 + 2))
            composite_plot(tit, xxl, yyl, zzl,
                    "Distance from TSS " + str(1 + kk) + " (bp)",
                    "Distance from TSS " + str(2 + kk) + " (bp)", textlat)
            savefig(figcomp, "{}_{}_{}_{}".format("K562", kk, ix, tit))
            plt.show()
```

```
0.5161772643586043
K562_0_987_RNA
**** 0
**** 1
```

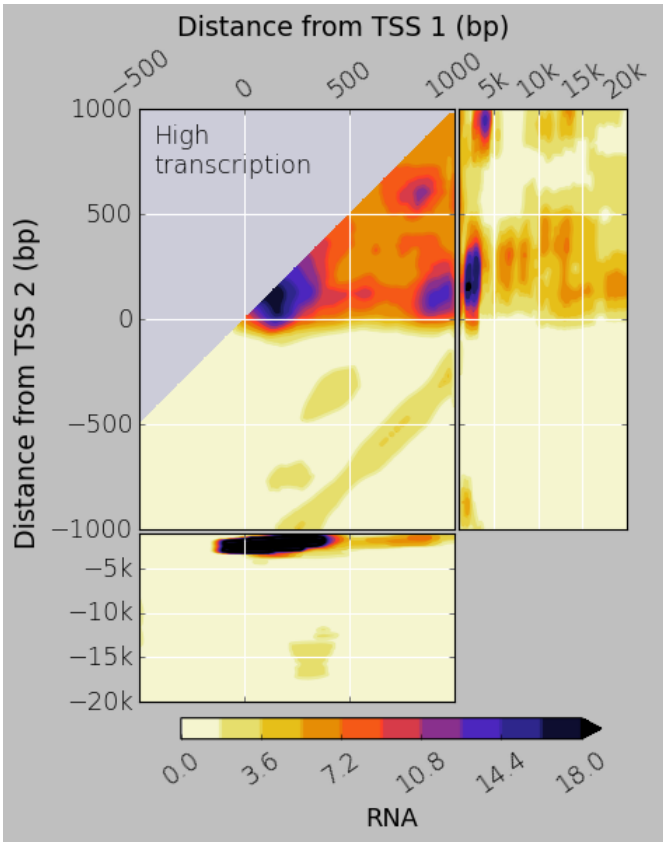


```
0.7681263861041233
K562_0_987_RAMPAGE
**** 0
**** 1
```

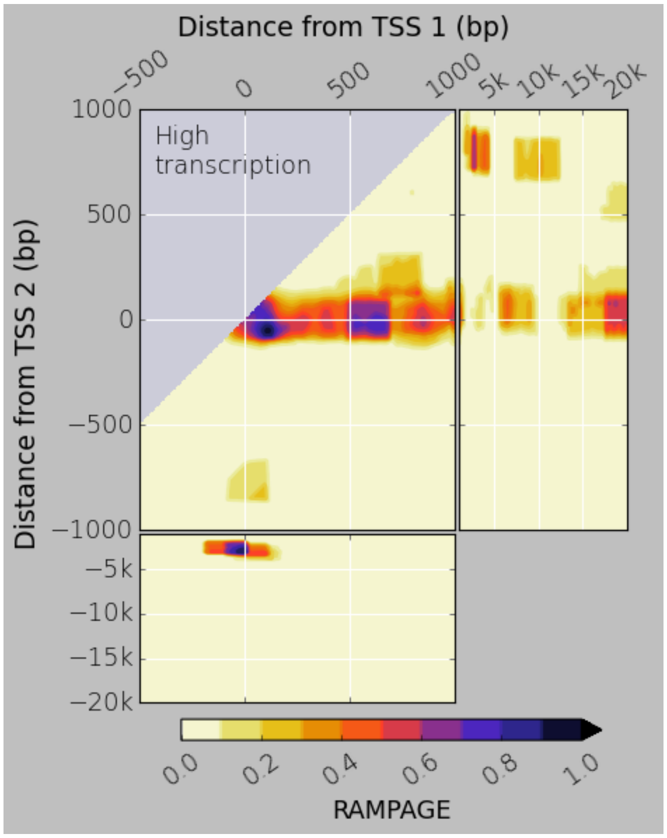


```
0.9710042200286878
K562_0_987_POLR2A
**** 0
**** 1
```

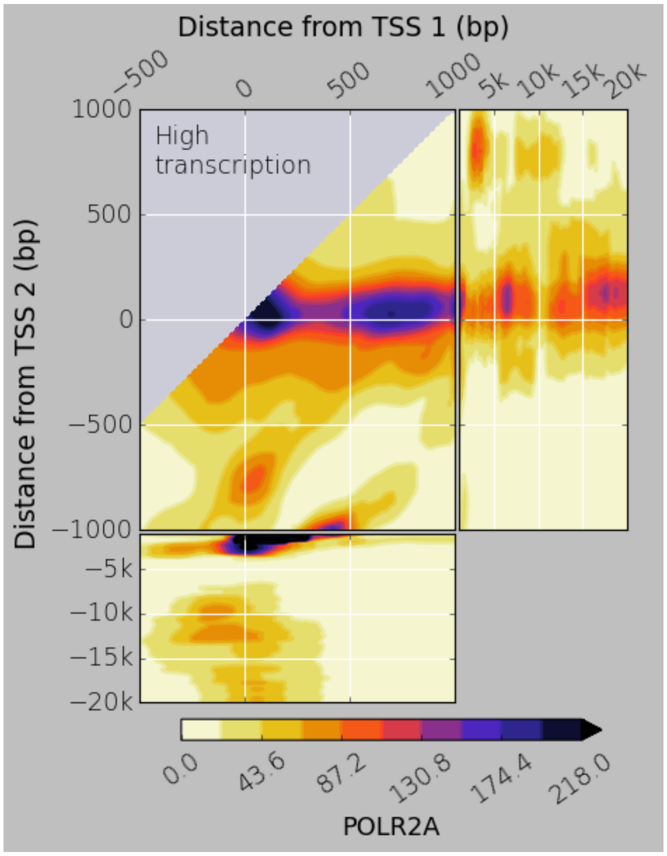

1.2687416567391838
K562_0_987_DNase
**** 0
**** 1

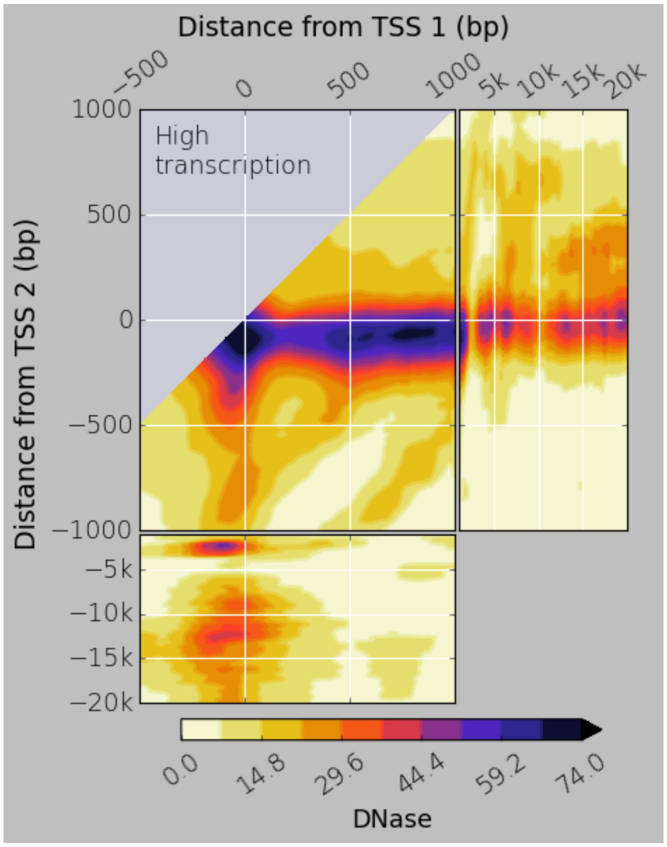

2.9496722864283242
K562_0_987_H3K4me3
**** 0
**** 1

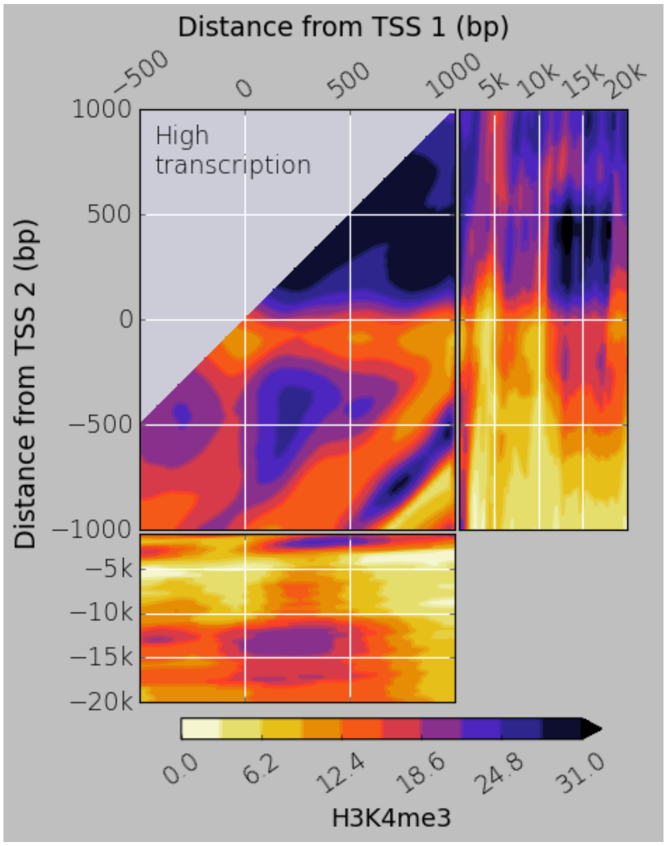

0.5161772643586043
K562_0_6107_RNA
**** 0
**** 1

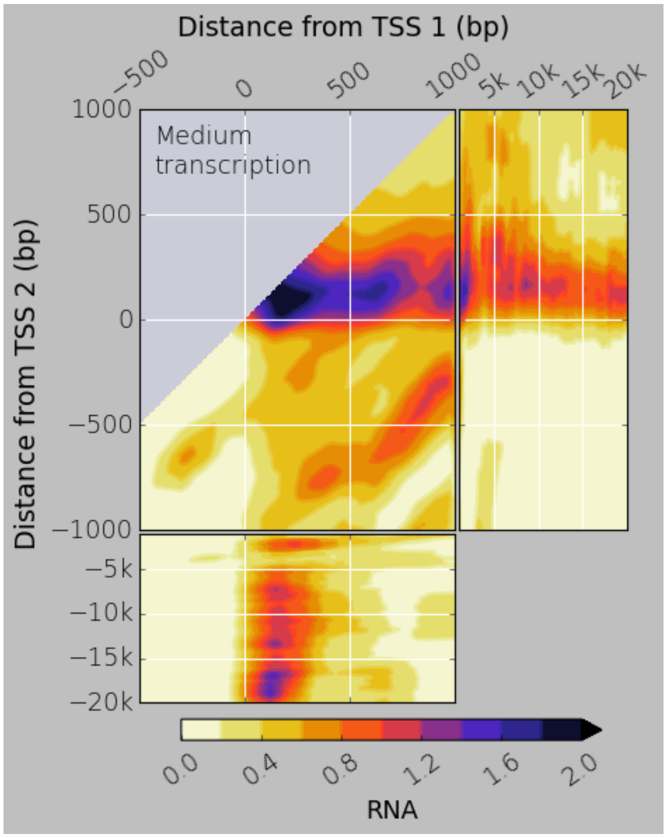

0.7681263861041233
K562_0_6107_RAMPAGE
**** 0
**** 1

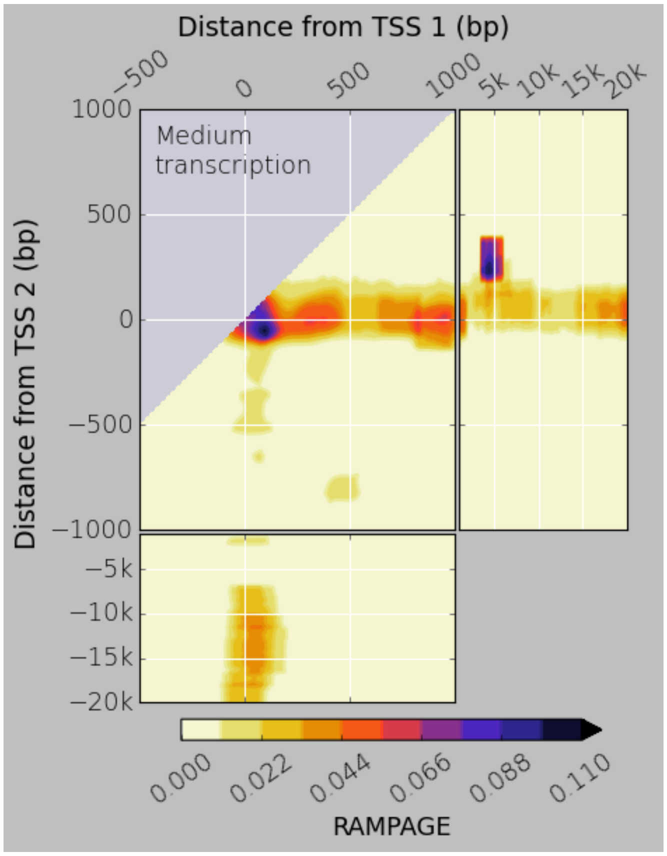

0.9710042200286878
K562_0_6107_POLR2A
**** 0
**** 1

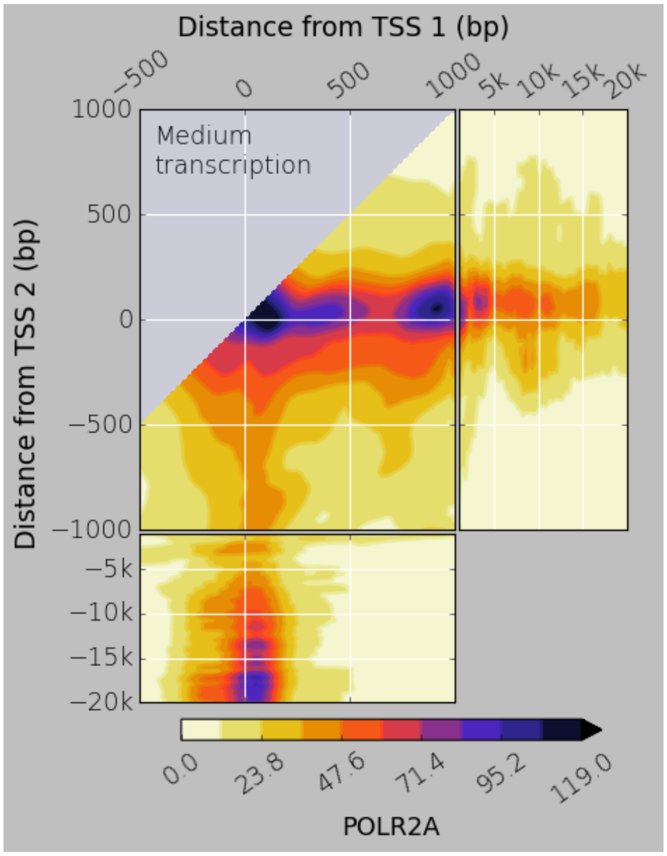

1.2687416567391838
K562_0_6107_DNase
**** 0
**** 1

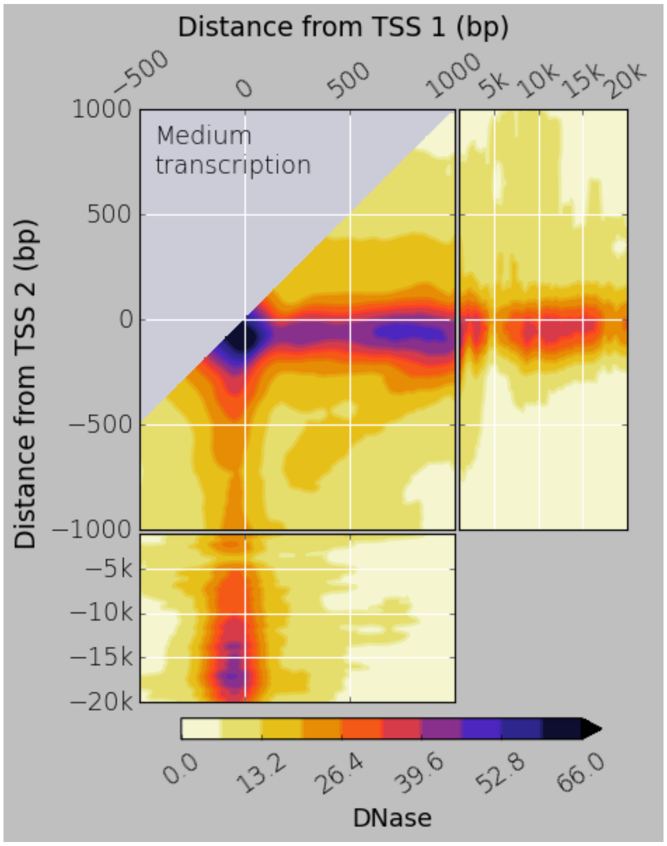


```
2.9496722864283242
K562_0_6107_H3K4me3
**** 0
**** 1
```

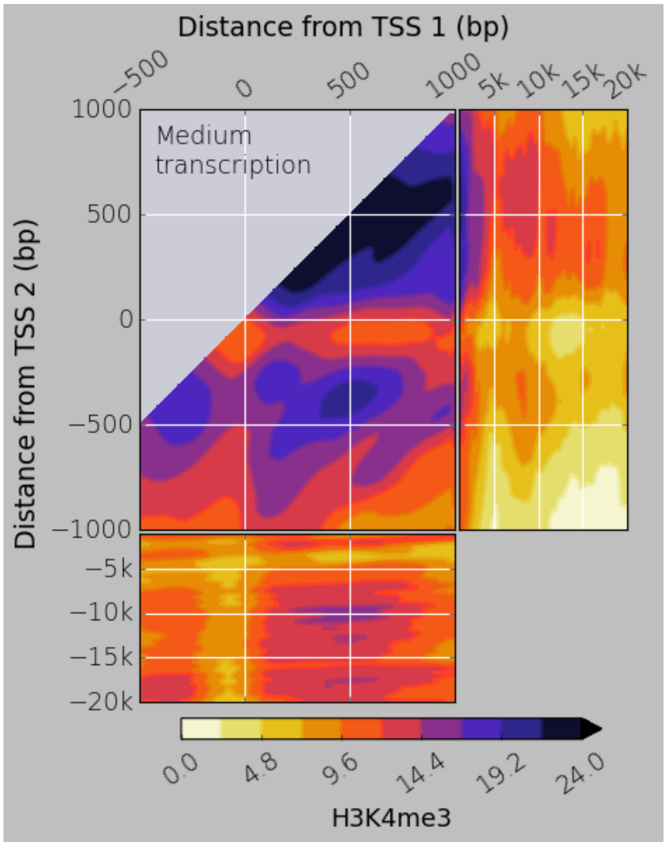


```
0.5161772643586043
K562_0_13641_RNA
**** 0
**** 1
```

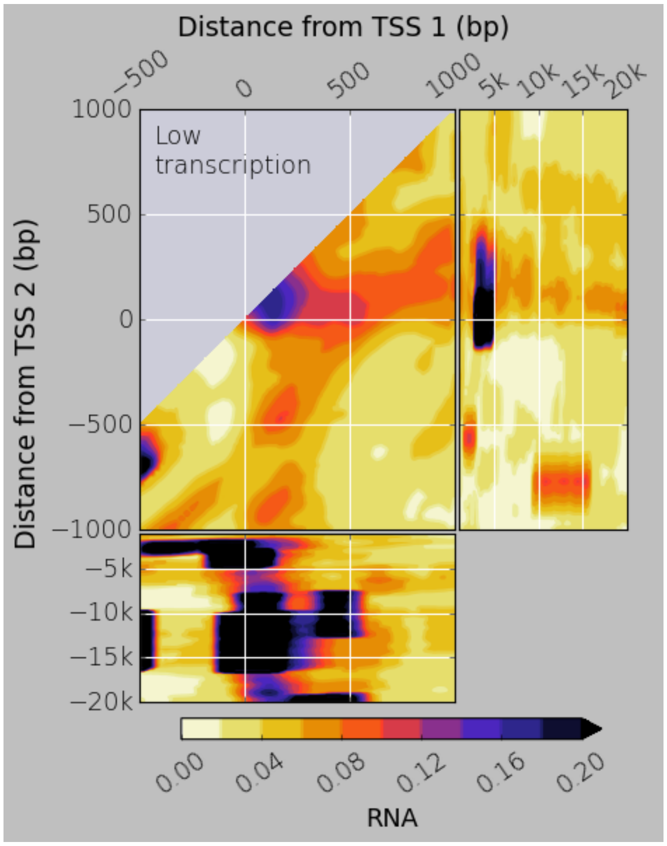

0.7681263861041233
K562_0_13641_RAMPAGE
**** 0
**** 1

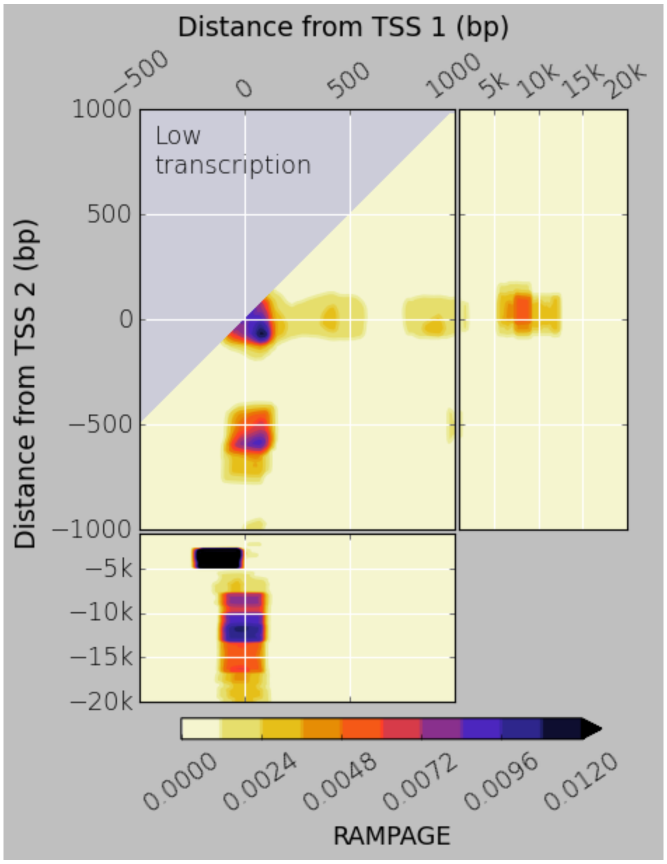

0.9710042200286878
K562_0_13641_POLR2A
**** 0
**** 1

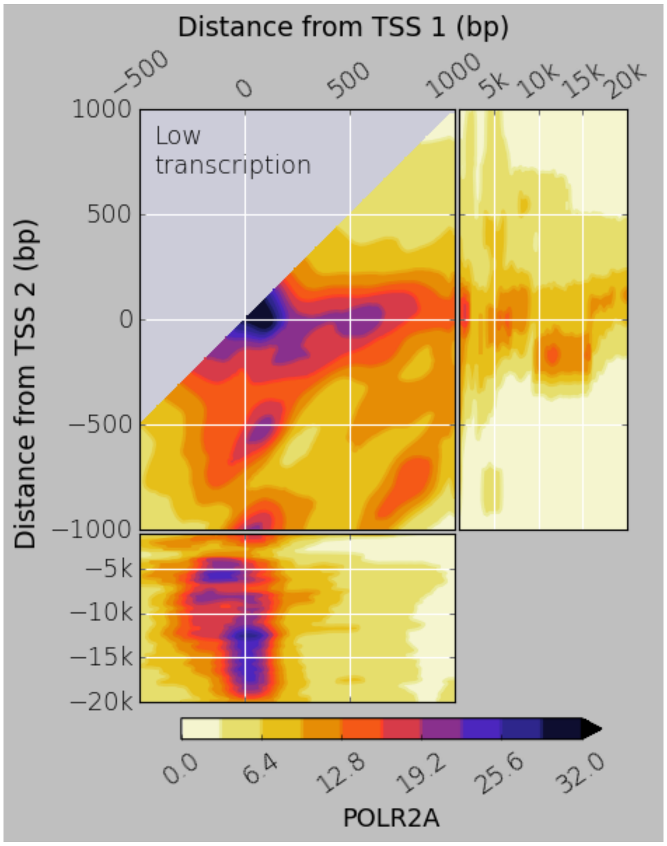

1.2687416567391838
K562_0_13641_DNase
**** 0
**** 1

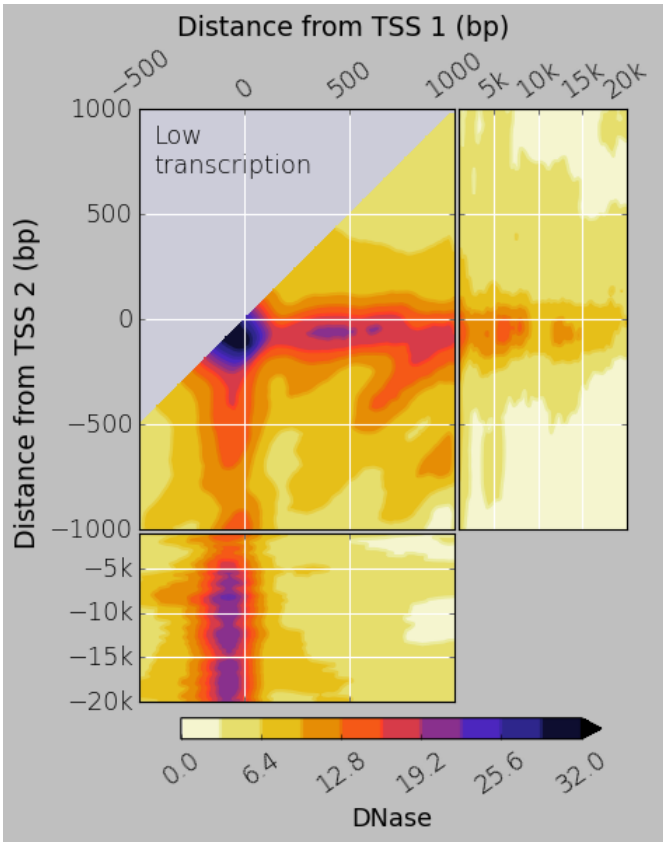

2.9496722864283242
K562_0_13641_H3K4me3
**** 0
**** 1

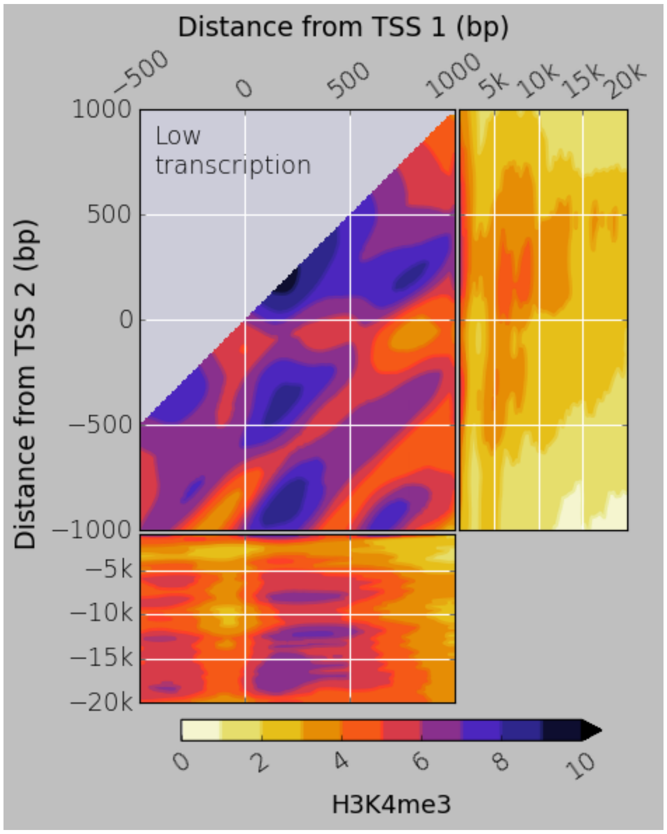

0.5161772643586043
K562_1_987_RNA
**** 1
**** 2

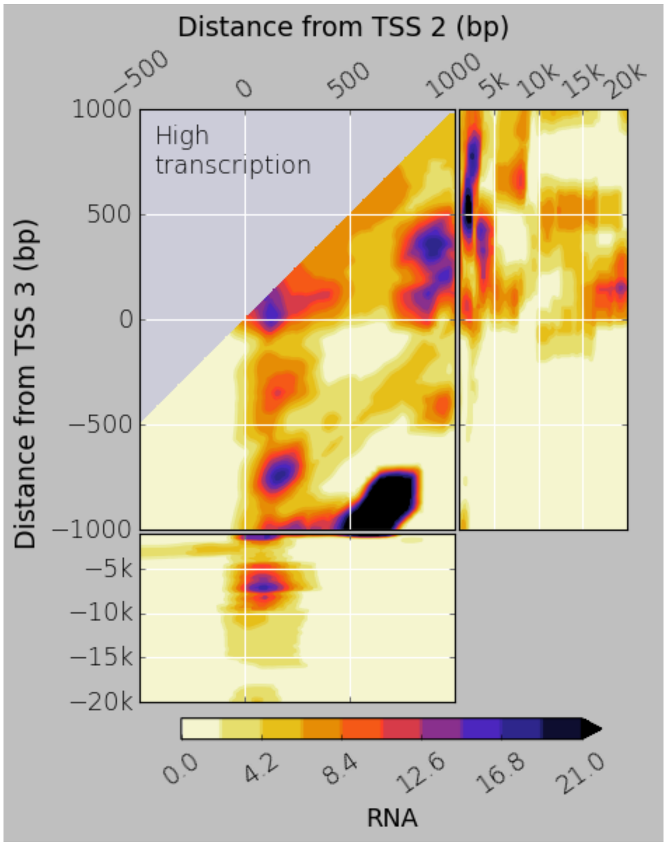

0.7681263861041233
K562_1_987_RAMPAGE
**** 1
**** 2

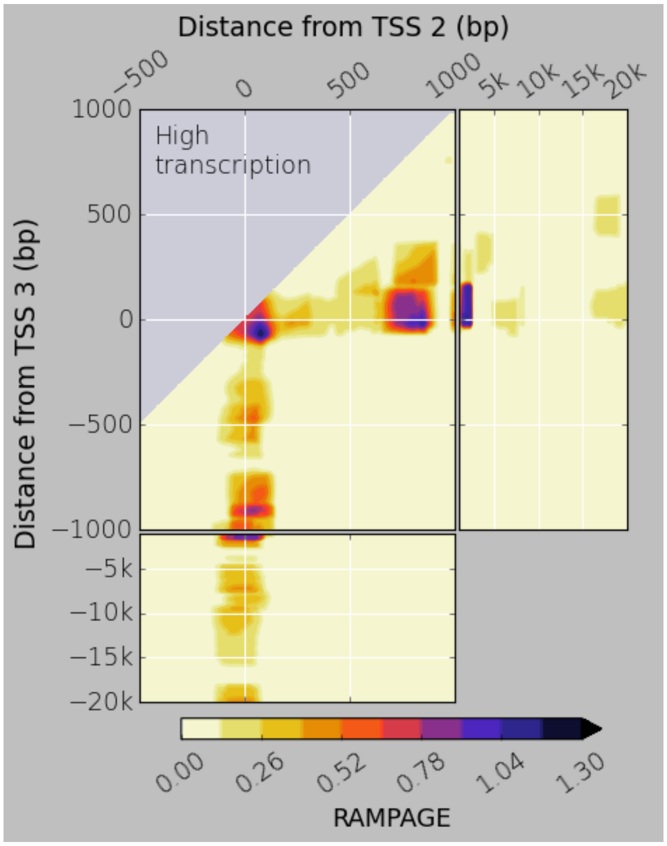


```
0.9710042200286878
K562_1_987_POLR2A
**** 1
**** 2
```

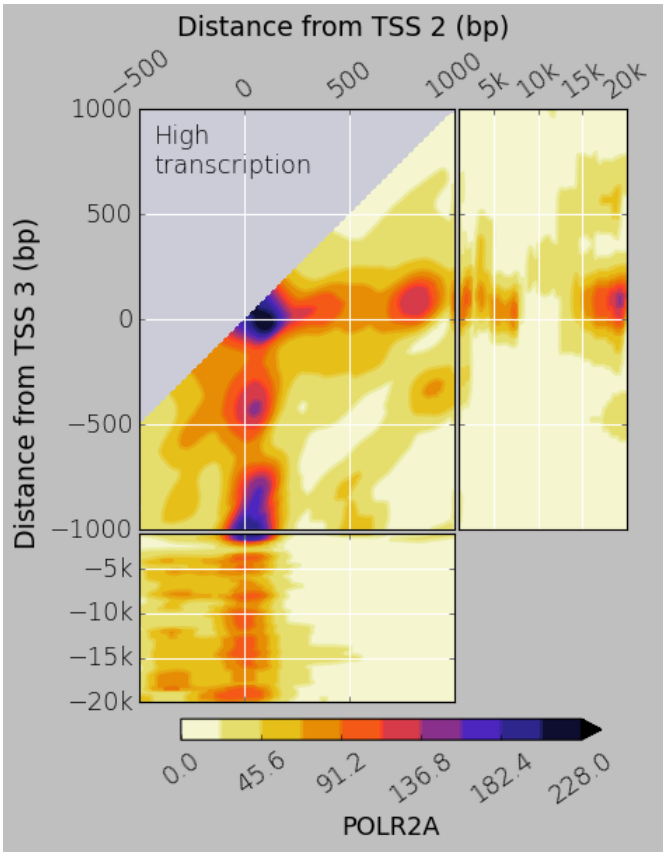


```
1.2687416567391838
K562_1_987_DNase
**** 1
**** 2
```

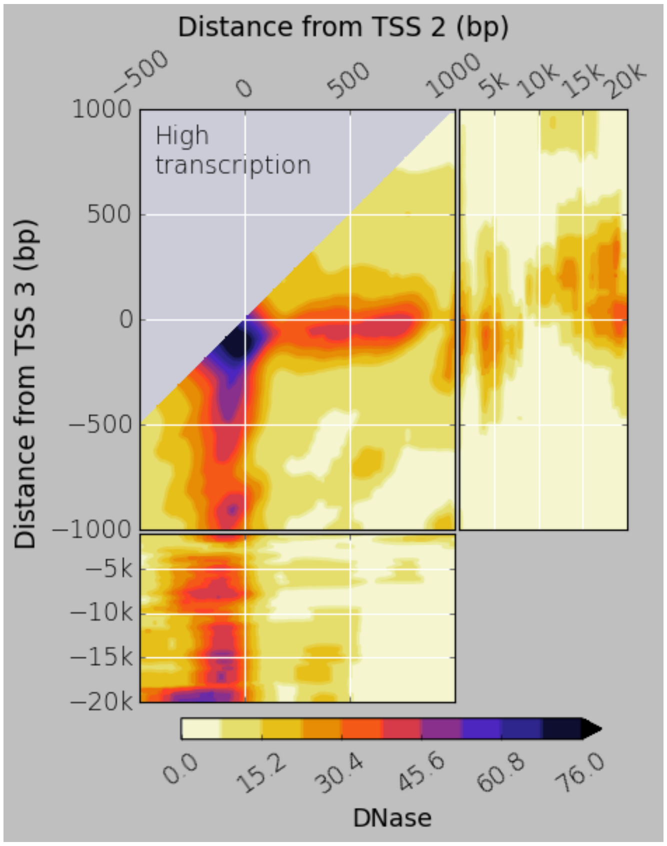


```
2.9496722864283242
K562_1_987_H3K4me3
**** 1
**** 2
```

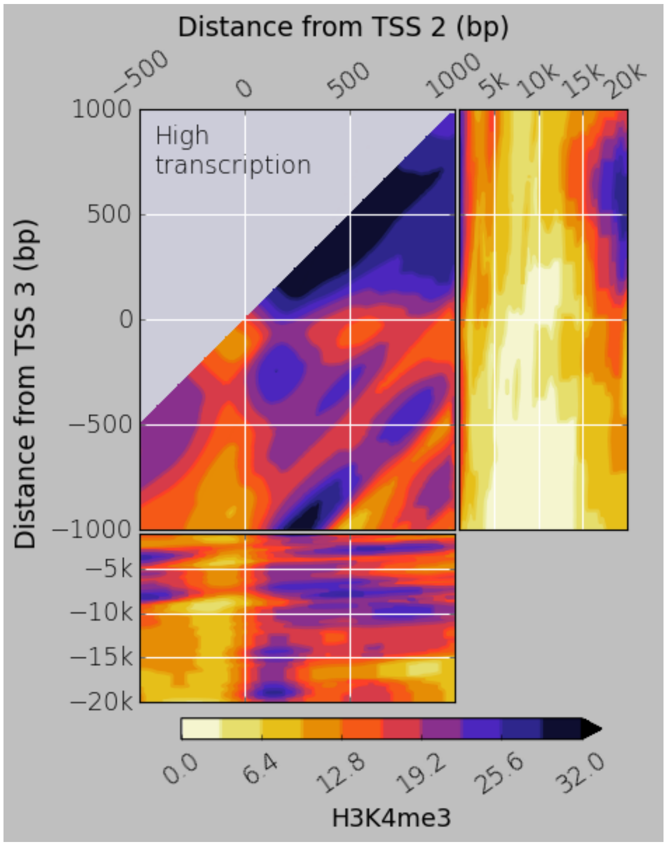


```
0.5161772643586043
K562_1_6107_RNA
**** 1
**** 2
```

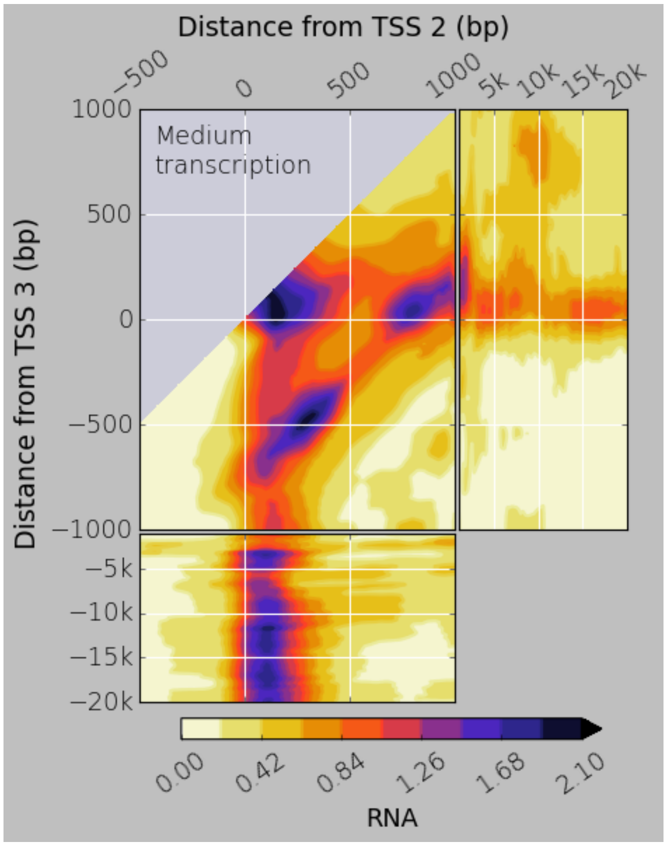

0.7681263861041233
K562_1_6107_RAMPAGE
**** 1
**** 2

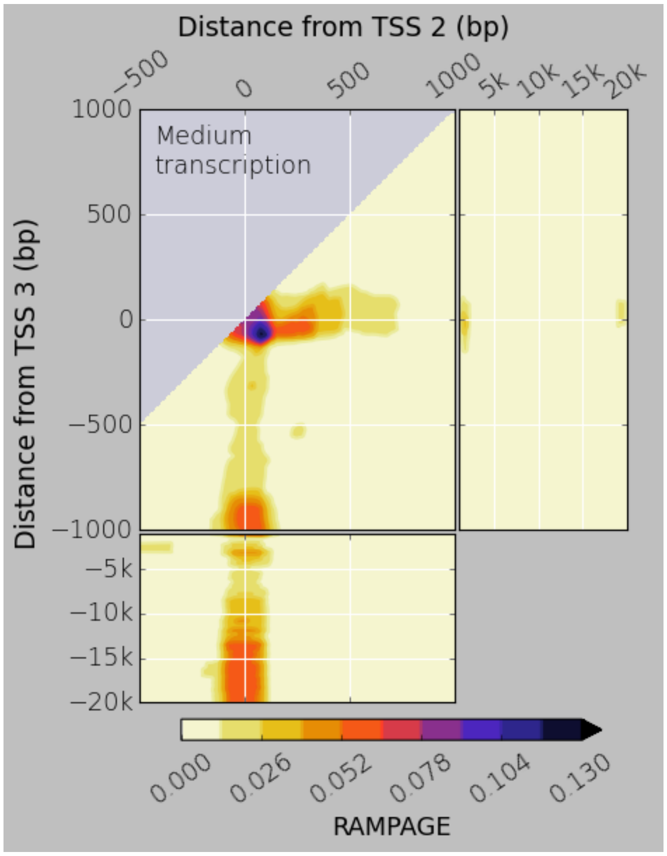

0.9710042200286878
K562_1_6107_POLR2A
**** 1
**** 2

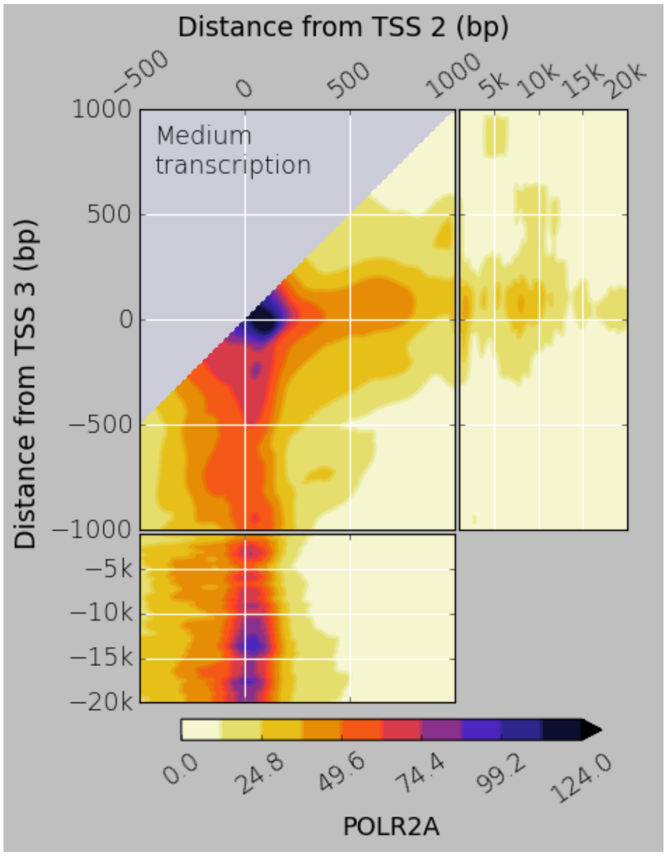

1.2687416567391838
K562_1_6107_DNase
**** 1
**** 2

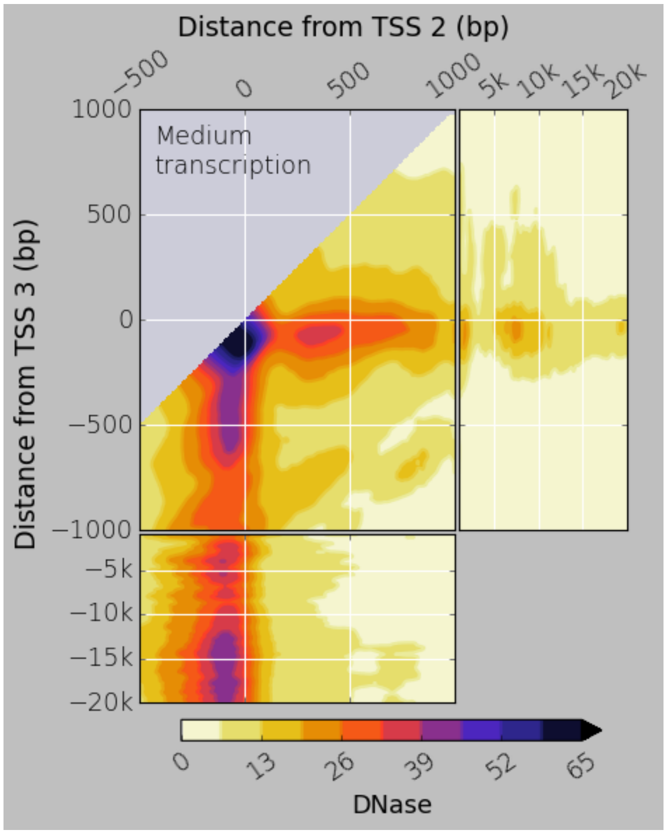

2.9496722864283242
K562_1_6107_H3K4me3
**** 1
**** 2

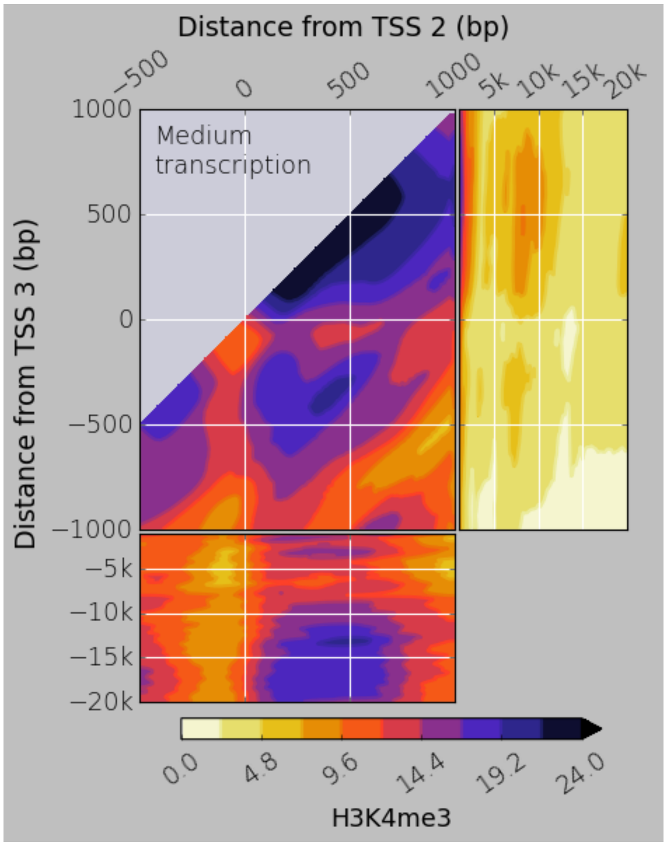

0.5161772643586043
K562_1_13641_RNA
**** 1
**** 2

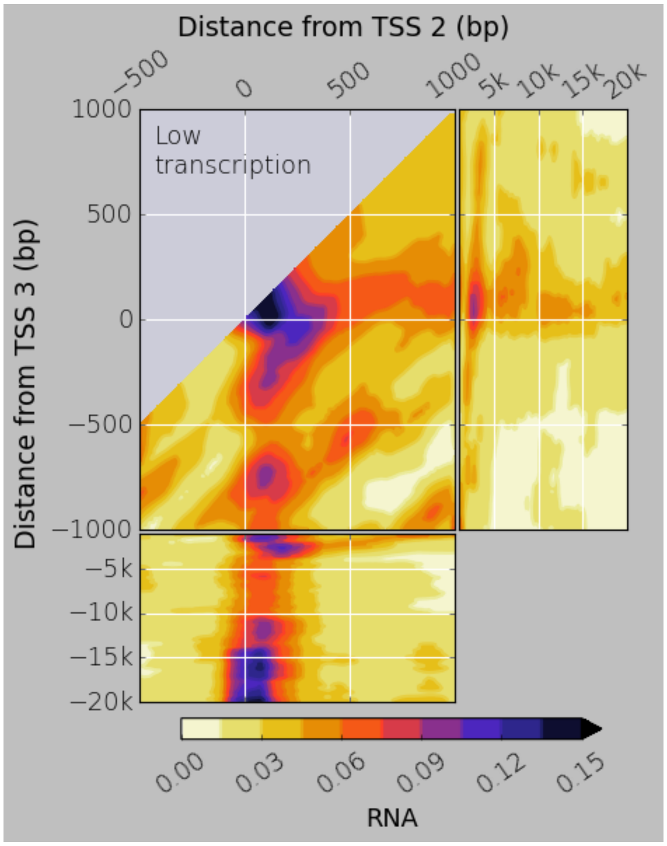

0.7681263861041233
K562_1_13641_RAMPAGE
**** 1
**** 2

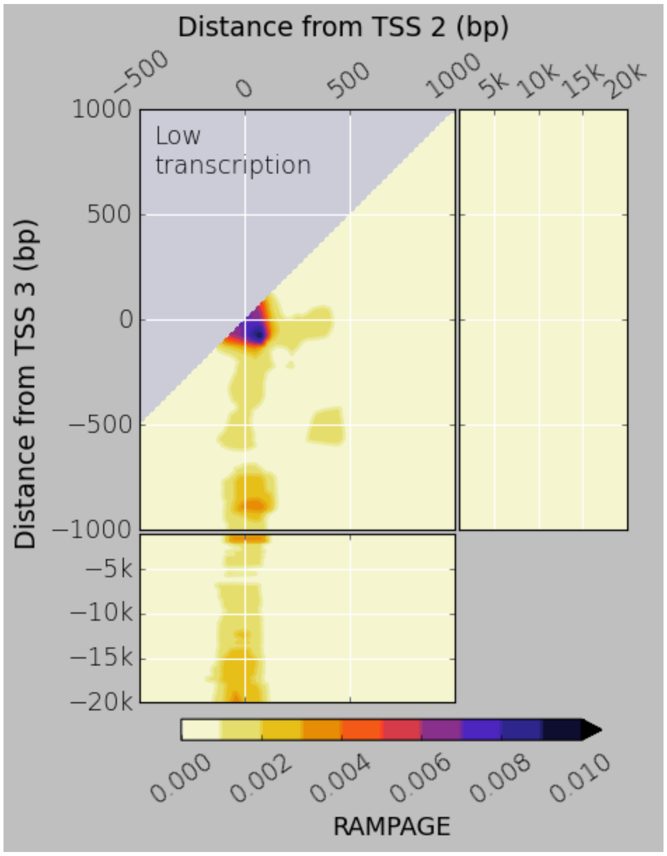

0.9710042200286878
K562_1_13641_POLR2A
**** 1
**** 2

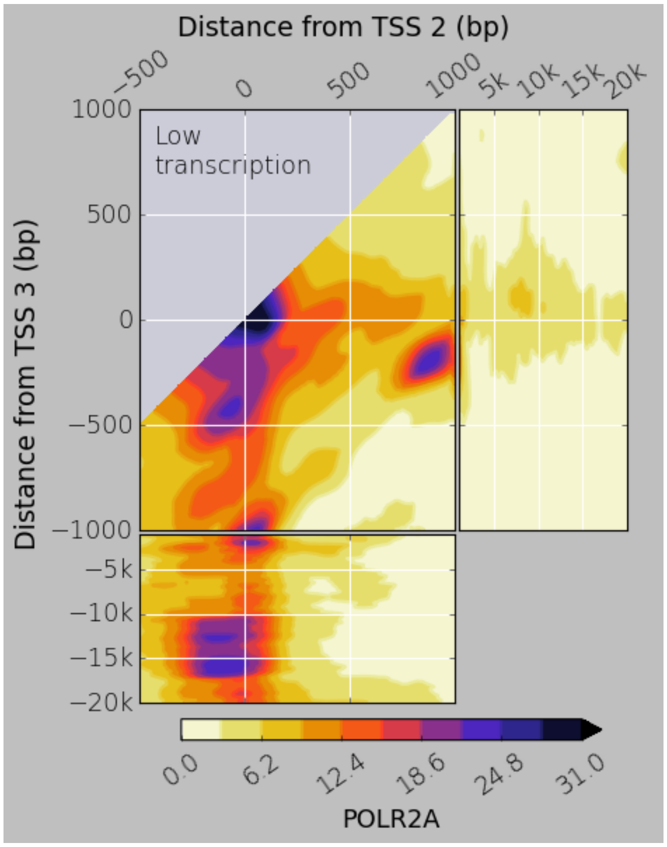

1.2687416567391838
K562_1_13641_DNase
**** 1
**** 2

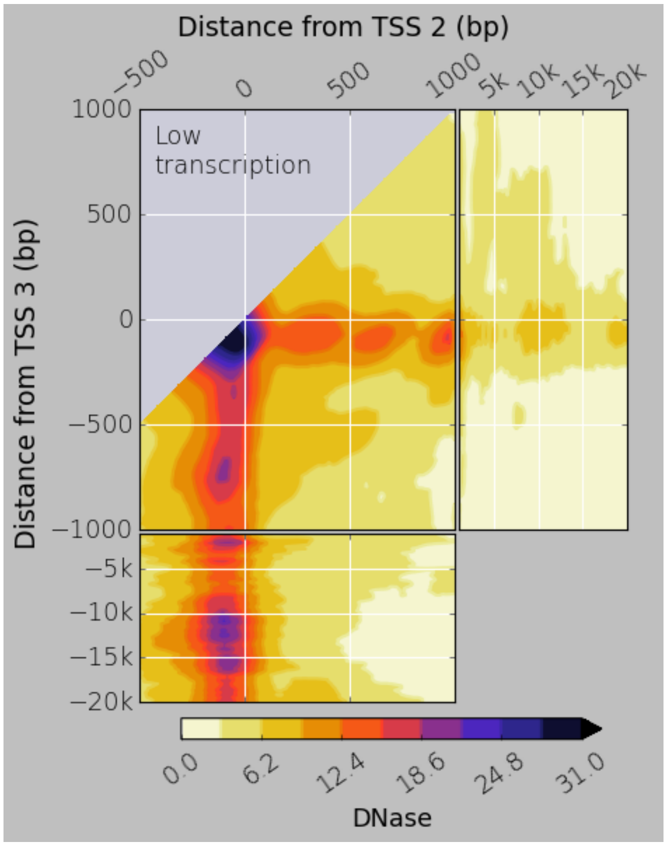


```
2.9496722864283242
K562_1_13641_H3K4me3
**** 1
**** 2
```

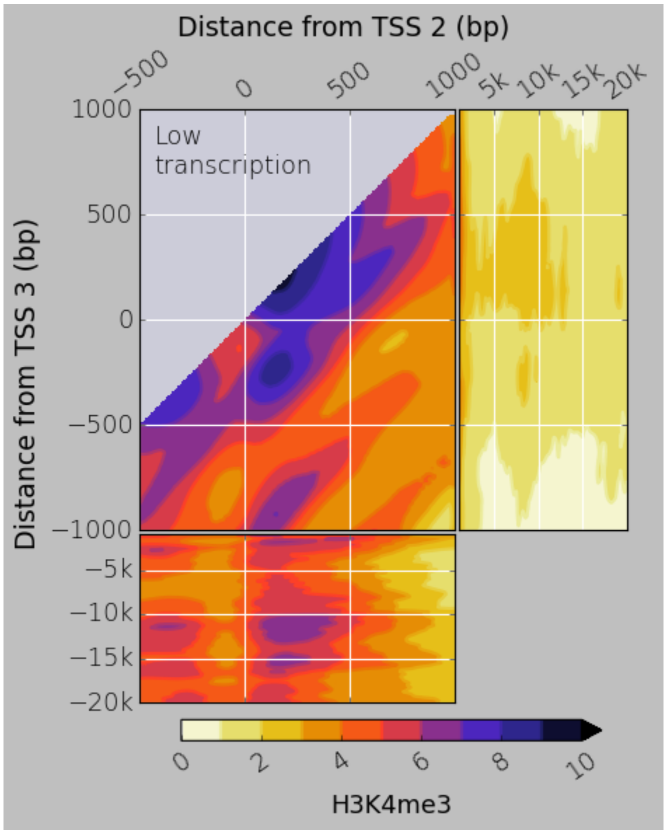

In [ ]:
