# Supplementary material for: Multi-landmark alignment of genomic signals reveals conserved expression patterns across transcription start sites
Source: Sci Rep. 2023 Jul 5;13:10835. doi: 10.1038/s41598-023-37140-x (PMC10322939; doi:10.1038/s41598-023-37140-x)
Supplement: Supplementary file 3 — Supplementary Information 3. [file 41598_2023_37140_MOESM3_ESM.pdf]

Supplementary Information

Supplementary figures

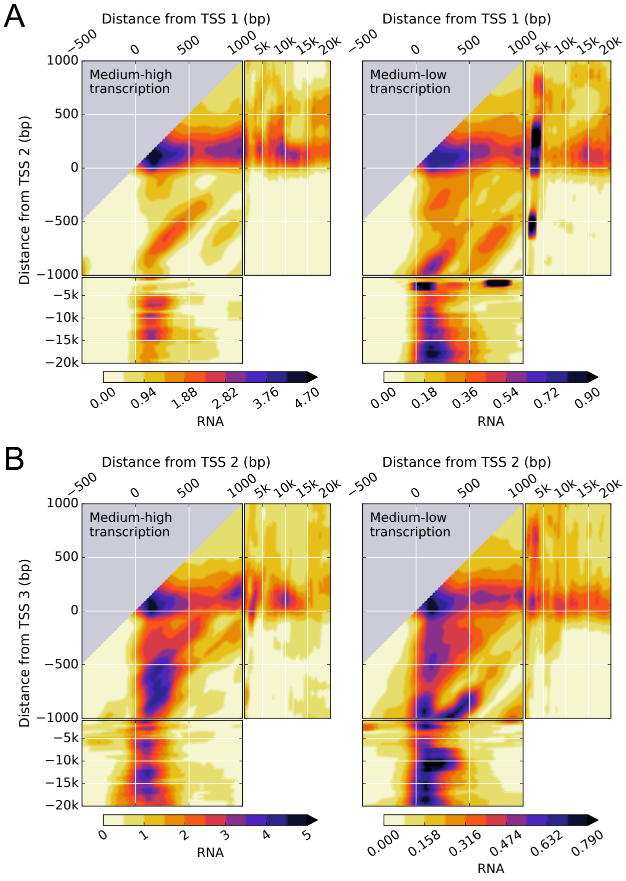

Figure S1: Transcription in K562 leukemia cell lines shows a complex dependence on the distance from pairs of TSSs, their intragenic position, and the transcriptional activity of the gene. (**A**, **B**), two-dimensional density of RNA-seq signal for pairs of the first (TSS 1) and second (TSS 2) TSSs (**A**) and the second (TSS 2) and third (TSS 3) TSSs (**B**) of genes with medium-high, and medium-low levels of transcription for the same conditions as in Fig. 2.

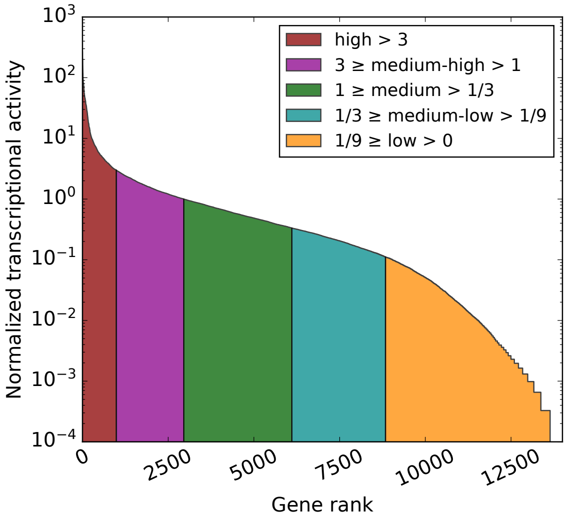

Figure S2: Normalized transcriptional activity (transcription / average transcription) of the protein coding genes in the K562 leukemia cell line ordered according to their transcriptional activity. The transcriptional activity of the gene was stratified as high, medium-high, medium, medium-low, and low. The number of genes in each category is 987, 1967, 3150, 2735, 4802, and 6685, respectively, of a total of 20327 protein-coding genes. Genes with zero transcriptional activity (zero RNA-seq read counts) are not considered. The ENCODE accession number for the gene quantifications is ENCFF782PCD.

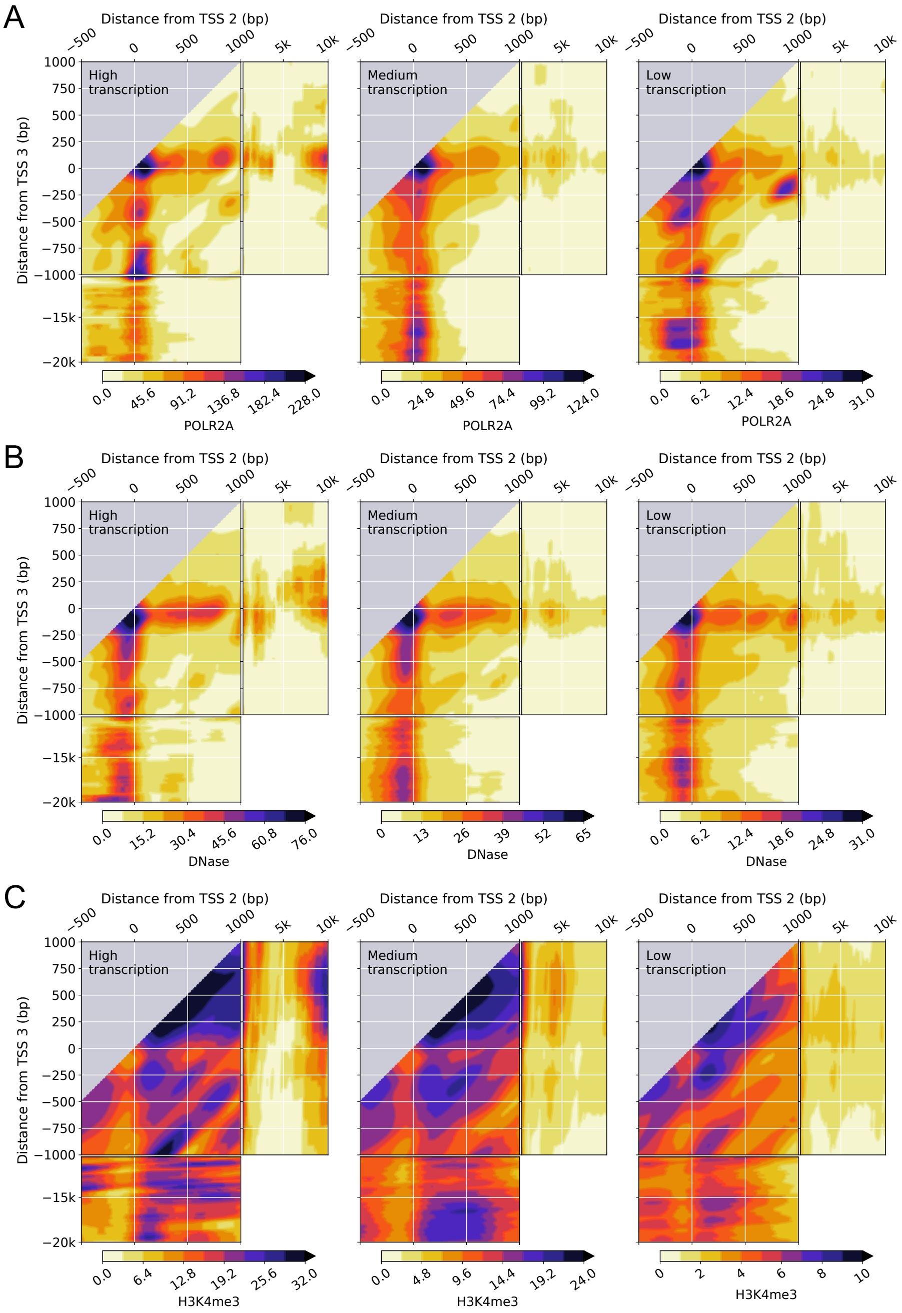

Figure S3: The intragenic position of the pair of TSSs affects the dependence of the RNA polymerase II occupancy, DNA accessibility, and H3K4me3 epigenetic chemical modification of the histone H3 protein on the distance from pairs of TSSs and the transcriptional activity of the gene in K562 leukemia cell lines. (**A**, **B**, **C**), two-dimensional density of POLR2A ChIP-seq signal (**A**), DNase-seq signal (**B**), H3K4me3 ChIP-seq signal (**B**) as in Fig. 4 but for pairs of the second (TSS 2) and third (TSS 3) TSSs instead of for pairs of the first (TSS 1) and second (TSS 2) TSSs.

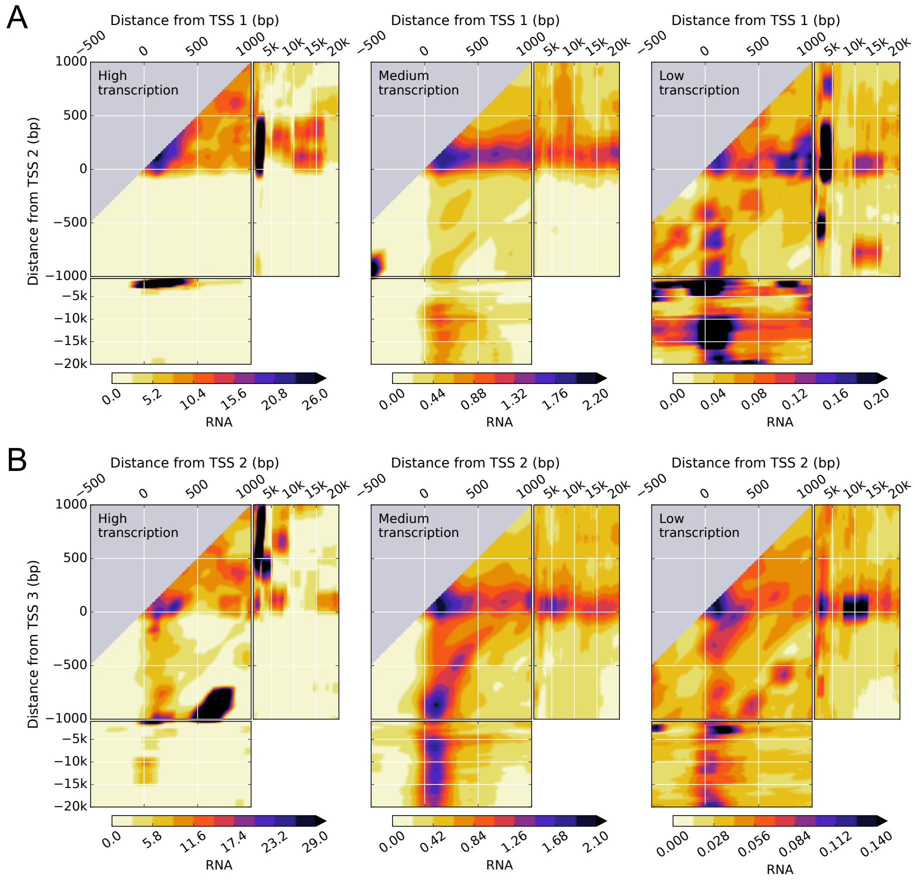

Figure S4: Transcription in GM12878 human lymphoblastoid cell line shows a complex dependence on the distance from pairs of TSSs, their intragenic position, and the transcriptional activity of the gene. (**A**, **B**), two-dimensional density of RNA-seq signal for pairs of the first (TSS 1) and second (TSS 2) TSSs (**A**) and the second (TSS 2) and third (TSS 3) TSSs (**B**) of genes with high, medium, and low levels of transcription. Data is available from the ENCODE consortium (experiment accession number ENCSR000AEC, Thomas Gingeras lab, CSHL). The accession numbers of the minus and plus strand RNA-seq signals and gene quantifications are ENCFF339IYH, ENCFF270HBR, and ENCFF610BLQ, respectively.

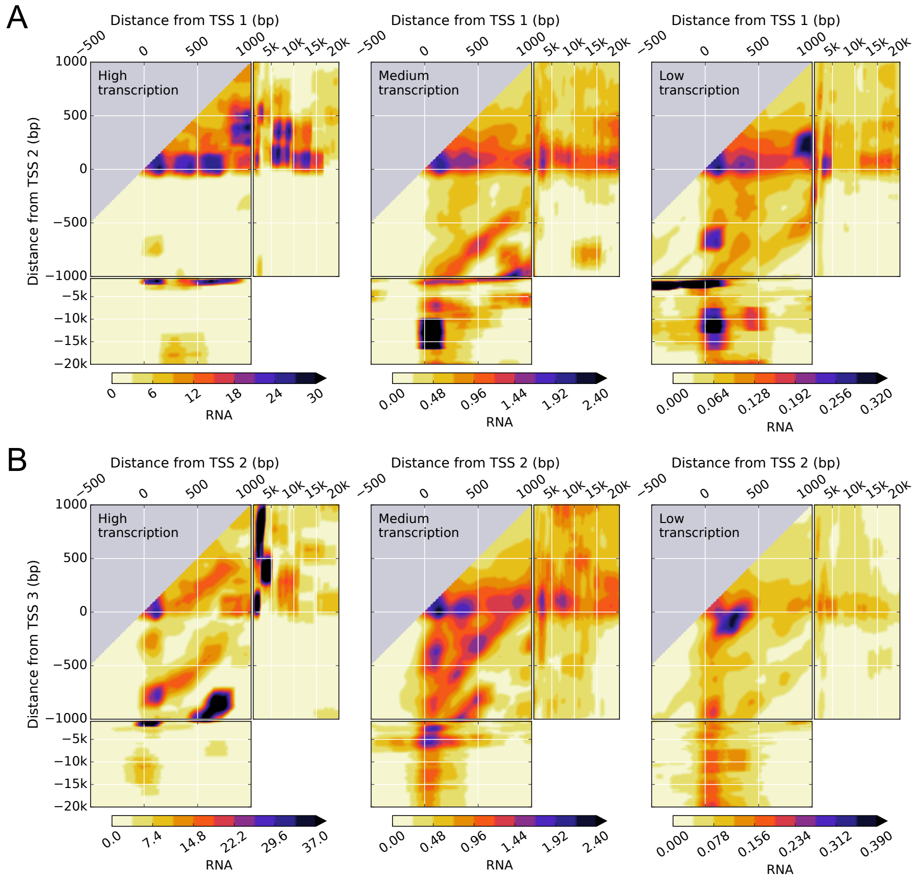

Figure S5: Transcription in H1-hESC human embryonic stem cells shows a complex dependence on the distance from pairs of TSSs, their intragenic position, and the transcriptional activity of the gene. (**A**, **B**), two-dimensional density of RNA-seq signal for pairs of the first (TSS 1) and second (TSS 2) TSSs (**A**) and the second (TSS 2) and third (TSS 3) TSSs (**B**) of genes with high, medium, and low levels of transcription. Data is available from the ENCODE consortium (experiment accession number ENCSR000COU, Thomas Gingeras lab, CSHL). The accession numbers of the minus and plus strand RNA-seq signals and gene quantifications are ENCFF587UUL, ENCFF587UUL, and ENCFF334LZL, respectively.

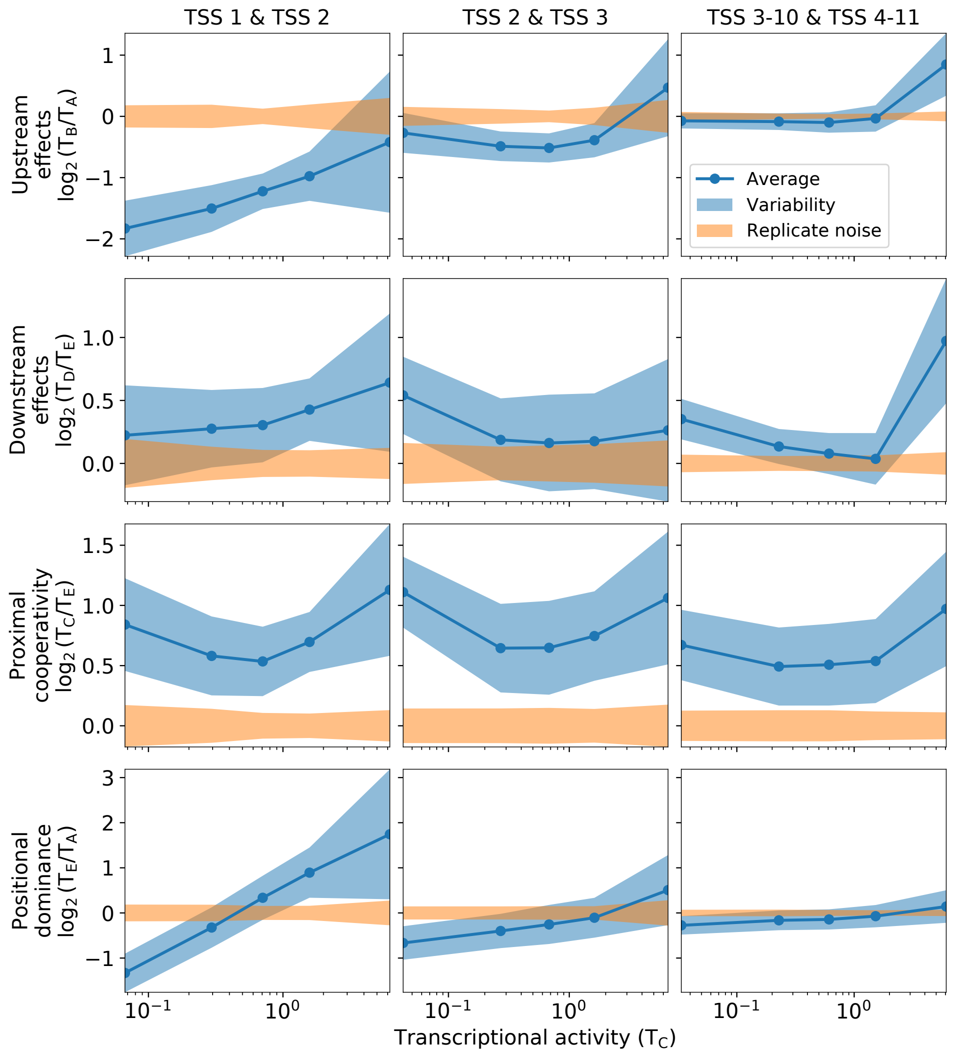

Figure S6: Average behavior, variability, and replicate noise of the interdependence of transcription at TSSs across human cell types. The statistical characterization of the interdependence is shown for the data in Fig. 5 for each of the five transcriptional levels. The blue lines represent the average of the $\log_2$ values over all the experiments; the blue-shaded region represents the variability computed as the $\pm$ standard deviation of the $\log_2$ replicate means of all the experiments; and the orange-shaded region represents the $\pm$ standard deviation of the replicate noise.

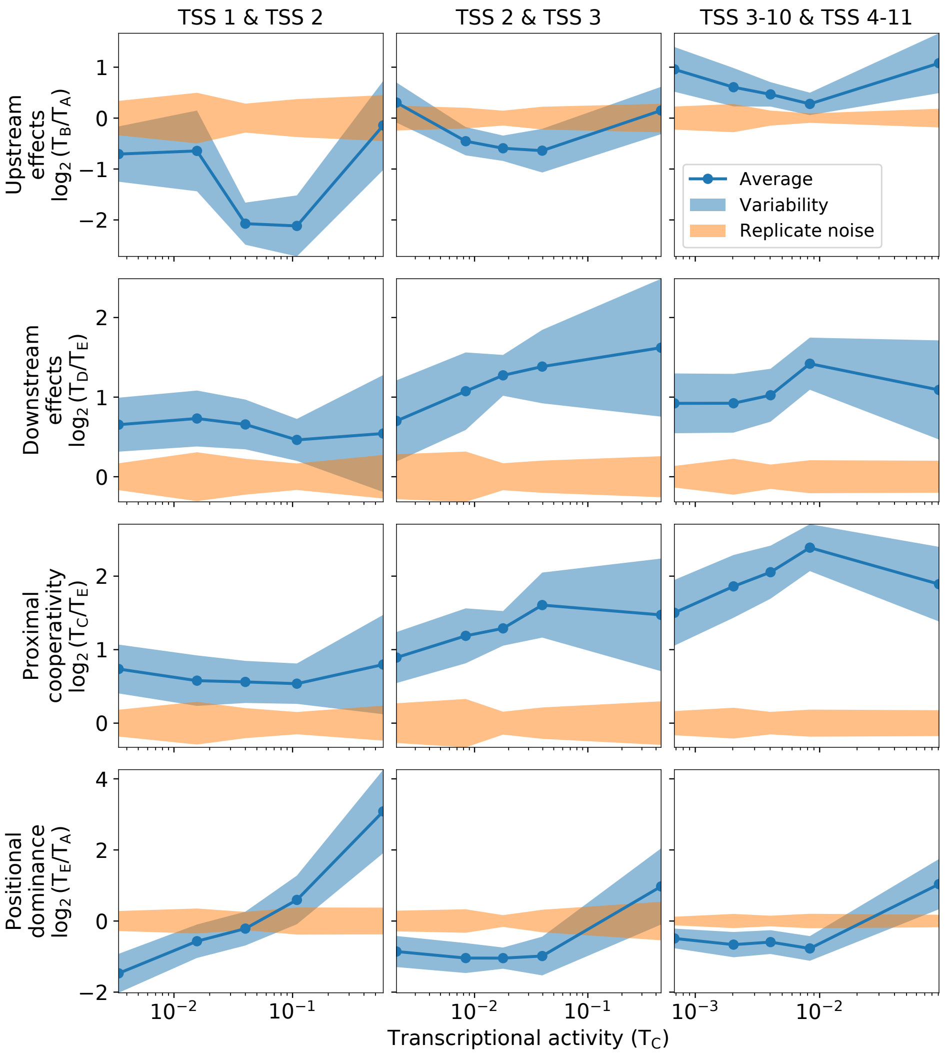

Figure S7: Average behavior, variability, and replicate noise of the interdependence of transcription initiation at TSSs across human cell types. The statistical characterization of the interdependence is shown for the data in Fig. 6 for each of the five transcriptional levels. The blue lines represent the average of the $\log_2$ values over all the experiments; the blue-shaded region represents the variability computed as the $\pm$ standard deviation of the $\log_2$ replicate means of all the experiments; and the orange-shaded region represents the $\pm$ standard deviation of the replicate noise.

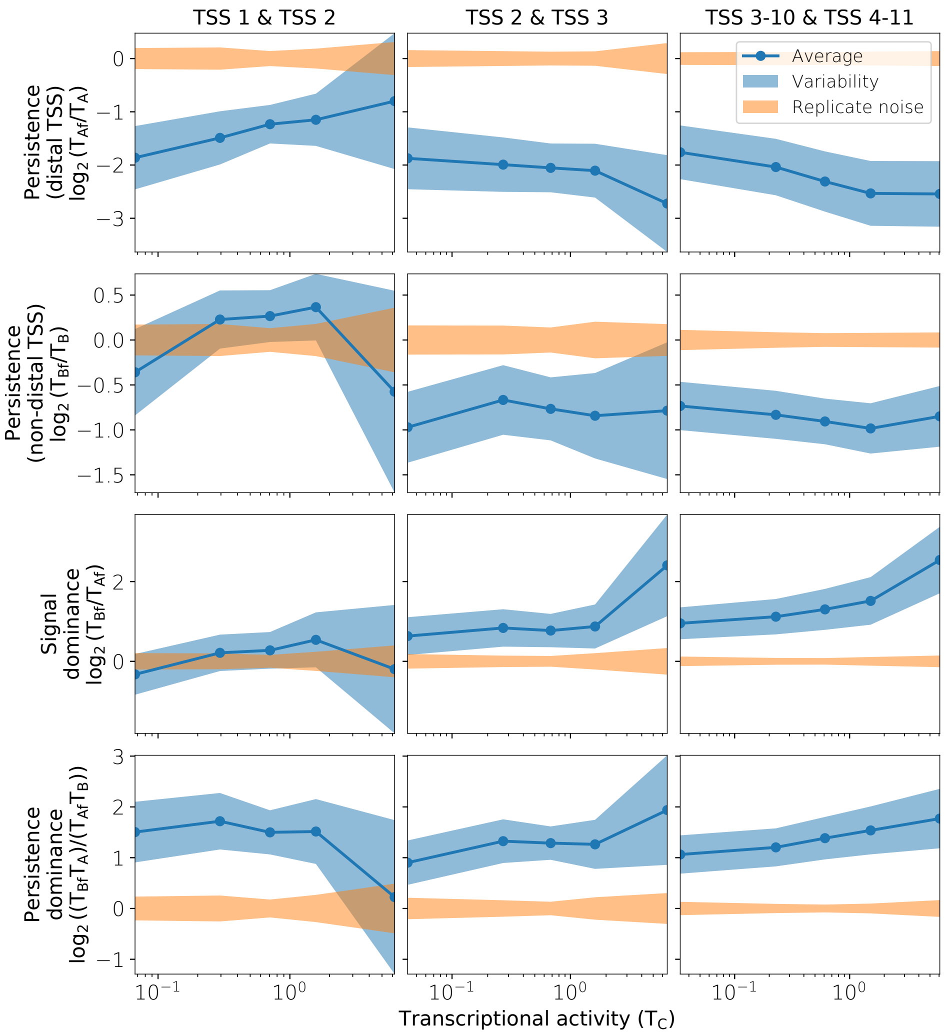

Figure S8: Average behavior, variability, and replicate noise of the interdependence of transcription between TSSs across human cell types. The statistical characterization of the interdependence is shown for the data in Fig. 7 for each of the five transcriptional levels. The blue lines represent the average of the $\log_2$ values over all the experiments; the blue-shaded region represents the variability computed as the $\pm$ standard deviation of the $\log_2$ replicate means of all the experiments; and the orange-shaded region represents the $\pm$ standard deviation of the replicate noise.

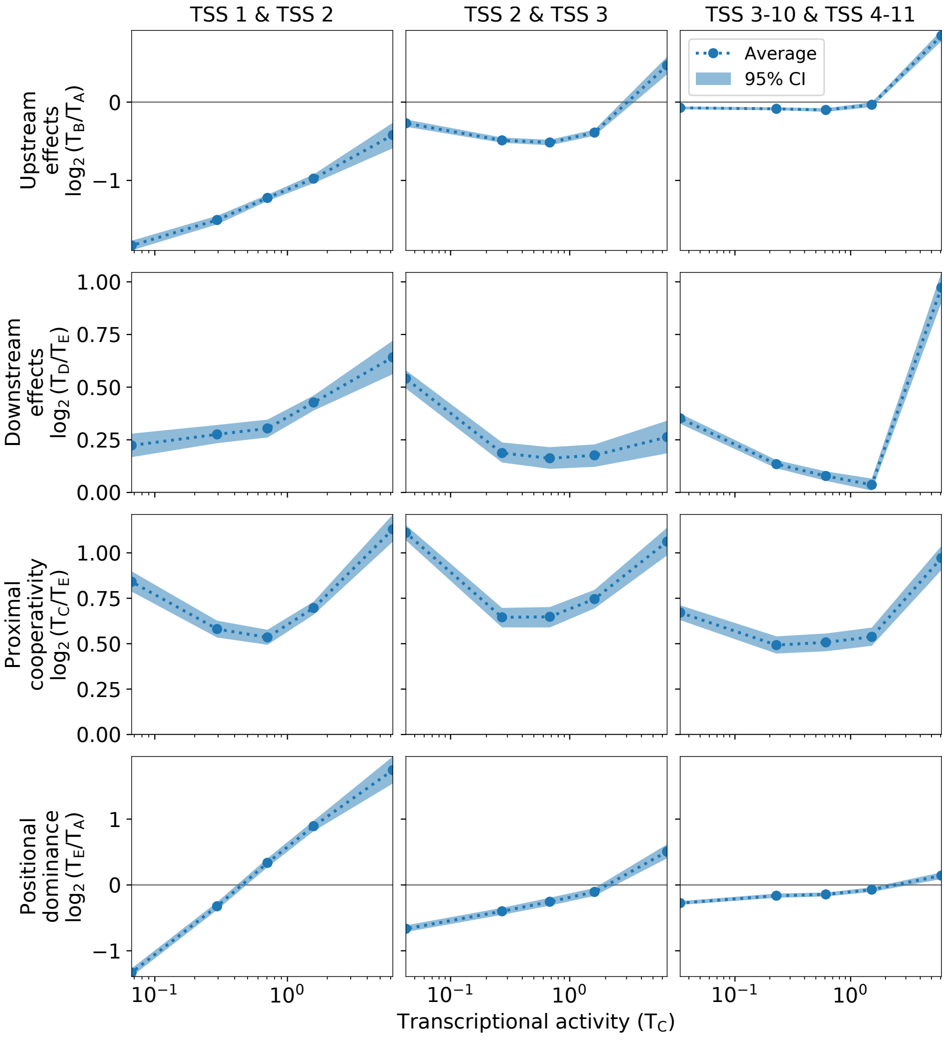

Figure S9: 95% confidence interval (CI) of the average interdependence of transcription at TSSs across human cell types. The statistical characterization of the interdependence is shown for the data in Fig. 5 for each of the five transcriptional levels. The blue lines represent the average of the $\log_2$ values over all the experiments and the blue-shaded region represents its 95% CI calculated using the bootstrap method.

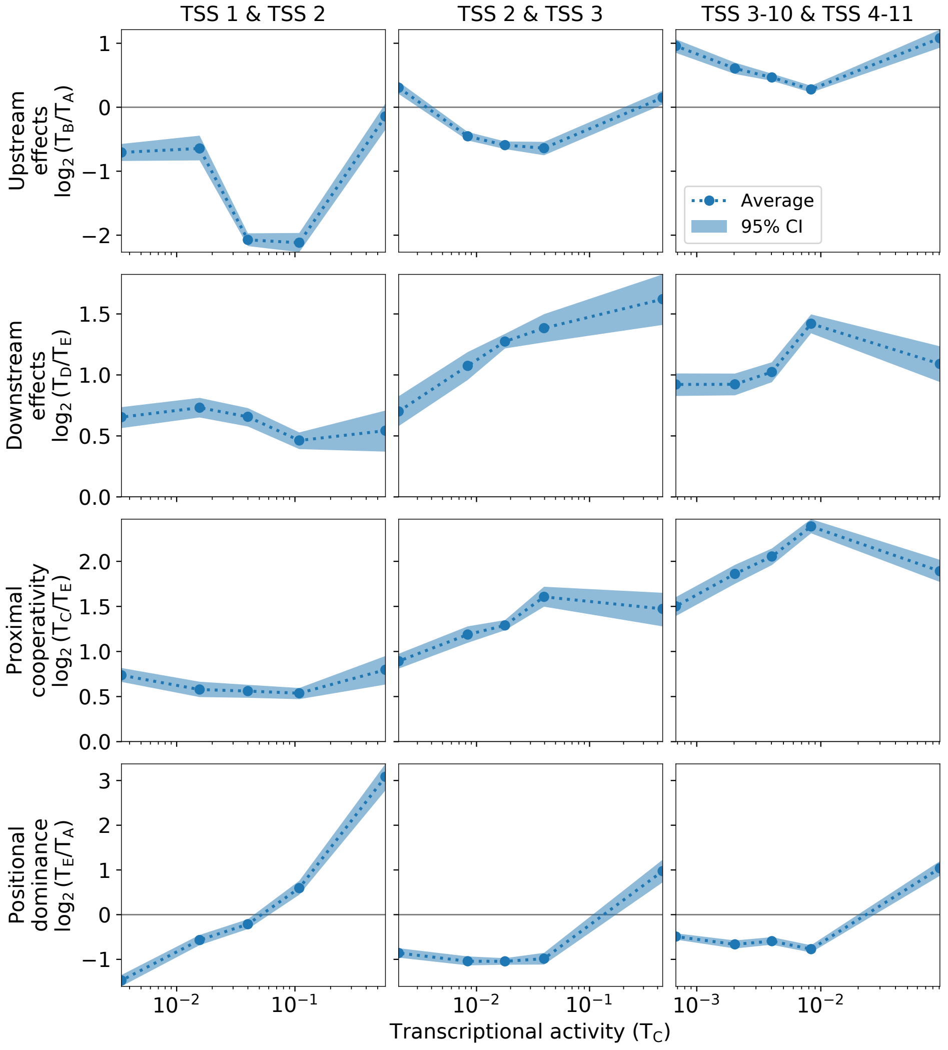

Figure S10: 95% confidence interval (CI) of the average interdependence of transcription initiation at TSSs across human cell types. The statistical characterization of the interdependence is shown for the data in Fig. 6 for each of the five transcriptional levels. The blue lines represent the average of the $\log_2$ values over all the experiments and the blue-shaded region represents its 95% CI calculated using the bootstrap method.

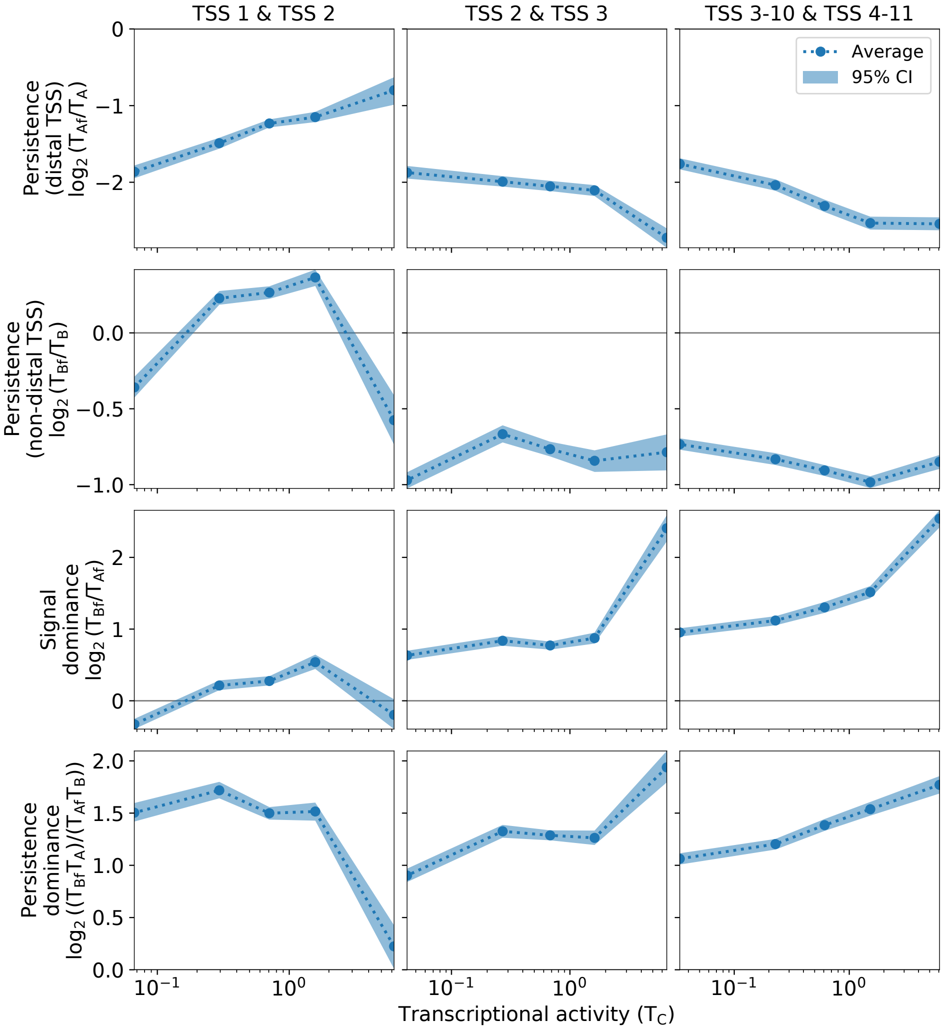

Figure S11: 95% confidence interval (CI) of the average interdependence of transcription between TSSs across human cell types. The statistical characterization of the interdependence is shown for the data in Fig. 7 for each of the five transcriptional levels. The blue lines represent the average of the $\log_2$ values over all the experiments and the blue-shaded region represents its 95% CI calculated using the bootstrap method.

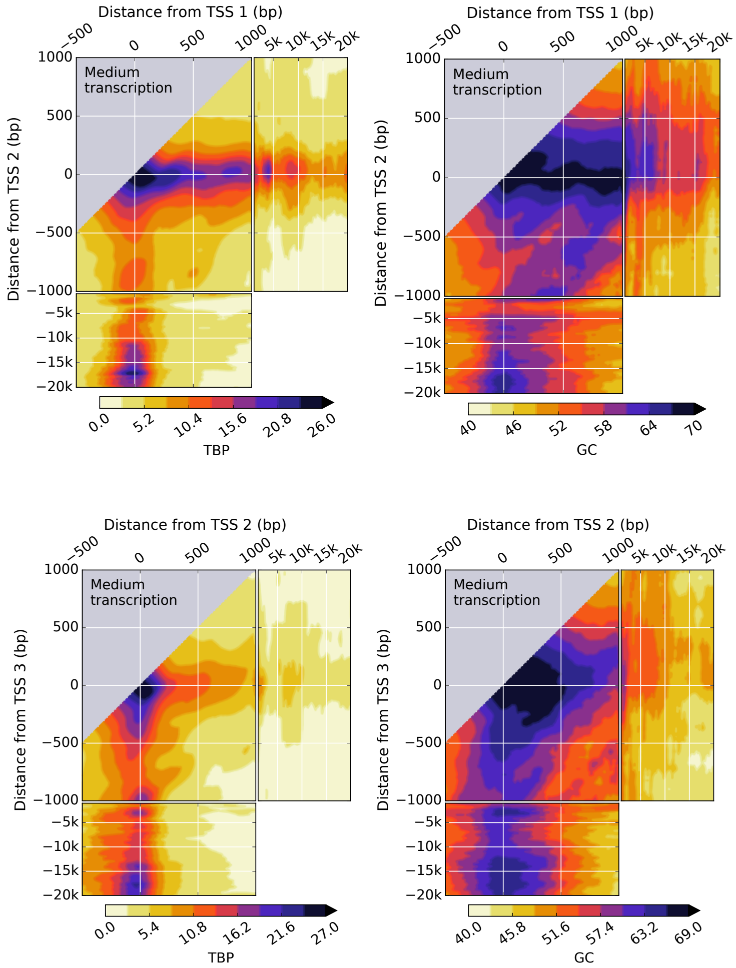

Figure S12: TATA-box binding protein (TBP) ChIP-seq and GC content correlate with the RNA-seq signal. Representative TBP ChIP-seq and % of GC content signal densities are shown for K562 leukemia cell lines.

Legends for supplementary tables

Table S1. Quantification of the average transcription in the seven representative regions of the two-dimensional signal density for different cell types for each of the pairs of consecutive TSSs up to the 11th TSSs in all human experiments in the ENCODE project with high replicate concordance. The data includes the experiment accession number, the biosample name, the biosample summary, the biosample type, the assay, the accession number for the plus strand, the accession number for the minus strand, the Spearman correlation between replicates, the replicate number, the stratified transcriptional activity level, the intragenic position of the TSS pair, and the corresponding average transcription in the regions A, Af, B, Bf, C, D, and E, labeled as T_A, T_Af, T_B, T_Bf, T_C, T_D, and T_E, respectively.

Table S2. Quantification of average transcription initiation performed in the same way as the quantification of average transcription in Table S1.

Data analysis software description

The data analysis was performed using custom Python 3.8 scripts implemented in Jupyter Notebooks available as supplemental files:

- 2DSignalDensities.ipynb. Notebook to compute two-dimensional signal densities, as in Figures 2, 3, and 4. It downloads the required bigWig signal files from ENCODE and the TSSs from Gencode.
- 2DRegionAverages.ipynb. Notebook detailing the code used to compute two-dimensional region averages, as in Supplementary Tables S1 and S2. It downloads the required bigWig signal files from ENCODE and the TSSs from Gencode.
